# HNRNPH1-stabilized LINC00662 promotes ovarian cancer progression by activating the GRP78/p38 pathway

**DOI:** 10.1038/s41388-021-01884-5

**Published:** 2021-06-19

**Authors:** Yong Wu, Qinhao Guo, Xingzhu Ju, Zhixiang Hu, Lingfang Xia, Yu Deng, Ping Zhao, Meng Zhang, Yang Shao, Shenglin Huang, Xianghuo He, Hao Wen, Xiaohua Wu

**Affiliations:** 1 Department of Gynecologic Oncology, Fudan University Shanghai Cancer Center, Shanghai, 200032 China; 2 Department of Oncology, Shanghai Medical College, Fudan University, Shanghai, 200032 China; 3 Fudan University Shanghai Cancer Center, Key Laboratory of Medical Epigenetics and Metabolism, Institutes of Biomedical Sciences, Fudan University, Shanghai, 200032 China; 4 Cancer Institute, Fudan University Shanghai Cancer Center, Shanghai, 200032 China; 5 Department of Pathology, The First Affiliated Hospital, Zhejiang University School of Medicine, Hangzhou, 310000 China; 6 Department of Pathology, Ruijin Hospital, Shanghai Jiaotong University School of Medicine, Shanghai, 200001 China; 7 Department of Pathology, Fudan University Shanghai Cancer Center, Shanghai, 200032 China

**Keywords:** Ovarian cancer, siRNAs

## Abstract

Numerous studies suggest an important role for copy number alterations (CNAs) in cancer progression. However, CNAs of long intergenic noncoding RNAs (lincRNAs) in ovarian cancer (OC) and their potential functions have not been fully investigated. Here, based on analysis of The Cancer Genome Atlas (TCGA) database, we identified in this study an oncogenic lincRNA termed LINC00662 that exhibited a significant correlation between its CNA and its increased expression. LINC00662 overexpression is highly associated with malignant features in OC patients and is a prognostic indicator. LINC00662 significantly promotes OC cell proliferation and metastasis in vitro and in vivo. Mechanistically, LINC00662 is stabilized by heterogeneous nuclear ribonucleoprotein H1 (HNRNPH1). Moreover, LINC00662 exerts oncogenic effects by interacting with glucose-regulated protein 78 (GRP78) and preventing its ubiquitination in OC cells, leading to activation of the oncogenic p38 MAPK signaling pathway. Taken together, our results define an oncogenic role for LINC00662 in OC progression mediated via GRP78/p38 signaling, with potential implications regarding therapeutic targets for OC.

## Introduction

Ovarian cancer (OC) comprises a heterogeneous group of tumors of several subtypes that exhibit distinct biological and molecular properties [1]. High-grade serous ovarian cancer (HGSOC) is the most common subtype and has a relatively poor prognosis due to very limited early-stage symptoms, widespread metastasis, and high relapse rate [2]. Despite substantial recent progress in our understanding of the genomics of OC, therapeutic approaches for patients have not been developed in parallel. Surgical debulking followed by chemotherapy remains the standard of care [3]. Thus, further exploration of the molecular mechanisms underlying OC pathogenesis and progression is urgently needed.

Current progress in transcriptomics has demonstrated that a major portion of the human transcriptome does not code for proteins [4]. Long intergenic noncoding RNAs (lincRNAs), recently defined as autonomously transcribed noncoding RNAs longer than 200 nucleotides that do not overlap annotated coding genes, share features with the other transcripts of the long noncoding RNA (lncRNA) family and constitute more than half of the lncRNA transcripts in the human transcriptome [5]. Accumulating evidence has demonstrated that lincRNAs participate in a wide range of cellular processes, including the regulation of epigenetic signatures and gene expression [5, 6]. Considering that lincRNAs, such as HOTAIR and LINC00992 have been named using different approaches, the present study focused on 294 lincRNAs with names containing the prefix “LINC”. In addition, The Cancer Genome Atlas (TCGA) project has elucidated the vital role of tumor molecular features in predicting patients’ prognosis and exploring potential driver target [7]. For instance, Wei Cao et al. constructed a three-lncRNA signature by analysis of a TCGA dataset, this signature may predict the prognosis of patients with head and neck squamous cell carcinoma [8]. This study aimed to identify an oncogenic lincRNA with prognostic value in a TCGA OC dataset.

Copy number alterations (CNAs), including deletions, insertions, duplications, and complex multisite variations ranging from 1000 bp to millions of bp, are widespread throughout the human genome [9]. CNAs can lead to dysregulated expression of genes, and multiple CNAs in the genome can cause heterogeneity in genomic and molecular phenotypes, leading to cancer occurrence and progression [10–12]. Previous studies of CNAs in tumors have more often investigated protein-coding genes than noncoding RNAs, and research on noncoding RNAs, especially lincRNA CNAs in ovarian tumors, is limited. Therefore, along with correlation analysis of lincRNA expression levels and CNAs, exploration of lincRNAs with dysregulation caused by abnormal CNAs is crucially important.

In the present study, we integrated the lincRNA CNA and transcriptome data of the dataset TCGA-OV and identified a lincRNA with copy number gain: LINC00662. LINC00662 is stabilized by heterogeneous nuclear ribonucleoprotein H1 (HNRNPH1) and exerts oncogenic biological effects by activating the glucose-regulated protein 78 (GRP78)/P38 signaling pathway in OC cells.

## Results

### LINC00662 is frequently upregulated in OC and positively associated with patient outcomes

**Fig. 1:**
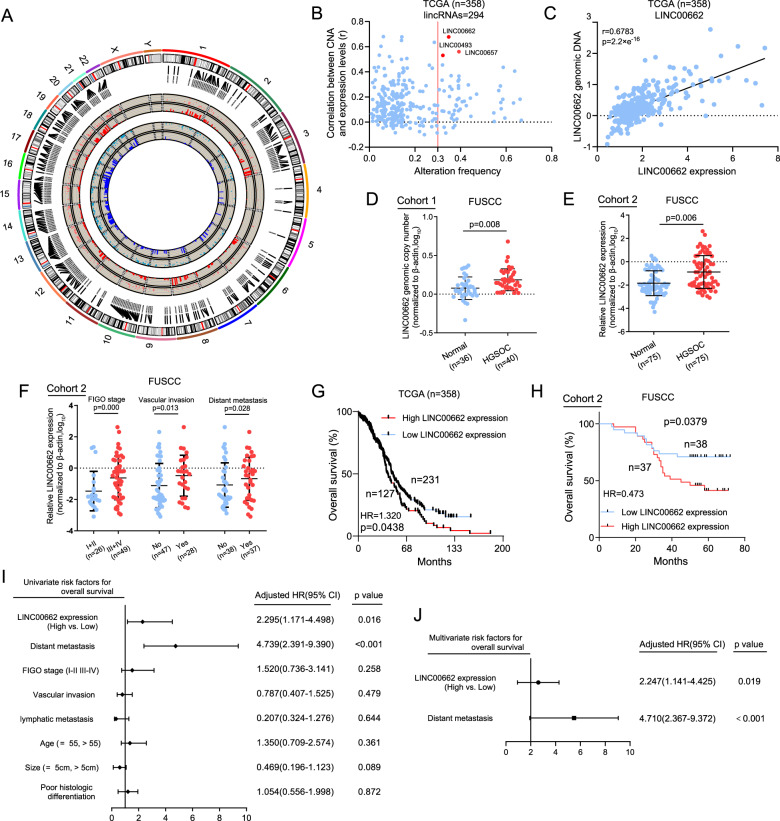
LINC00662 is overexpressed in OC and clinically associated with patient prognosis. **A** Circos plot showing the genome-wide profile of copy number variations in genes harboring the 294 lincRNAs with names including the prefix “LINC”. The outer ring sections indicate the chromosomes. The size of each section is relative to the size of that chromosome. The Circos plot is divided into two tracks: the histogram on the outer track displays the copy number amplifications of the lincRNAs; the histogram on the inner track shows the copy number deletions of the lincRNAs. The red bar indicates amplification and the blue bar deletion. **B** Alteration frequencies and correlations between CNAs and expression of lincRNAs in OC. Each dot represents one lncRNA gene locus. The red dots indicate that LINC00662, LINC00657, and LINC00493 were amplified, with expression significantly influenced by CNA. **C** A strong positive correlation between LINC00662 gene copy number and RNA expression was observed in 358 patients in the dataset from TCGA. **D** The copy numbers of LINC00662 were determined in 40 HGSOC tissues and 36 unpaired normal ovarian DNA samples collected from FUSCC using qRT-PCR. **E** RNA levels of LINC00662 in 75 unmatched OC and normal ovarian tissues. Log_10_ ratio dot plots were generated from data obtained by qRT-PCR analyses. **F** Clinical significance of LINC00662 in patients with OC. High LINC00662 expression correlated positively with advanced FIGO stage and distant metastasis. **G** OS curves of OC patients with high and low LINC00662 RNA expression levels from TCGA. **H** Kaplan–Meier plots of OC patients stratified by LINC00662 expression level. **I**, **J** Univariate analysis and multivariate analysis showed that LINC00662 was an independent factor for OC prognosis. Bars correspond to 95% confidence intervals.

To investigate the role of CNAs of lincRNAs in ovarian tumorigenesis, we cross-referenced a list of lincRNAs in the published literature to compile a comprehensive list of 294 lincRNAs prefixed with “LINC”. First, CNA profiles for 358 patients in the TCGA-OV dataset were analyzed (Fig. 1A). A total of 55 significant lincRNAs were identified, corresponding to an alteration frequency >30% among all OC samples (Fig. 1B, Supplementary Table S1). Second, considering that genomic alterations are often accompanied by dysregulated expression of genes, these 55 lincRNAs were ranked according to correlations between their CNAs and RNA expression levels (Supplementary Table S1). Among the 55 lincRNAs, the three highest-ranked lincRNAs (LINC00662, LINC00657, and LINC00493) were identified as candidate genes (Fig. 1B). Third, overall survival (OS) analysis was performed with Kaplan–Meier Plotter (http://kmplot.com/analysis/index.php?p=service), and the results showed that LINC00662 was significantly correlated with poor survival (*P* = 0.00027), whereas LINC00657 and LINC00493 had no prognostic predictive values for the OS of OC patients (*P* = 0.12, *P* = 0.15, Supplementary Fig. S1A–C). Thus, we selected LINC00662 as the candidate lincRNA for further study. A positive correlation with a confidence level of 0.95 was found between the genomic copy number and the RNA expression level of LINC00662 in the TCGA dataset (*r* = 0.6783, *P* < 0.001, Fig. 1C). Genomic copy number amplification of LINC00662 was further validated in Fudan University Shanghai Cancer Center (FUSCC) cohort 1, containing 40 HGSOC and 36 normal ovarian tissue DNA samples; the increase in its RNA level was validated in FUSCC cohort 2, containing 75 HGSOC and 75 normal ovarian samples (Fig. 1D, E). LINC00662, located on chromosome 19q11, contains 883 nucleotides and four exons, as determined via 5′ and 3′ RACE analyses (Supplementary Fig. S2A, B). In addition, analyses with CPC, CPAT indicated that LINC00662 is a nonprotein-coding lincRNA (Supplementary Fig. S2C, D). Subcellular fractionation and RT-PCR analyses showed that LINC00662 predominantly localizes in the cytoplasm of OC cells (Supplementary Fig. S2E), and this localization was also found by in situ hybridization (ISH; Supplementary Fig. S2F). To assess the clinical significance of LINC00662, the relationships between LINC00662 RNA levels and clinicopathological features were then analyzed. LINC00662 expression correlated positively with the International Federation of Gynecology and Obstetrics (FIGO) stage and distant metastasis (Fig. 1F). Importantly, high LINC00662 levels were markedly associated with poor OS in TCGA CNA dataset and Cohort 2 patients (Fig. 1G, H); however, the disease-free survival (DFS) of the groups with low and high LINC00662 levels did not differ, may owing to the limited samples (Supplementary Fig. S1D). In addition, univariate and multivariate (Fig. 1I, J) regression analyses demonstrated LINC00662 to be an independent predictor for OS in OC patients. These data suggest that LINC00662 might function as an oncogene in OC, owing to its considerable copy number amplification.

### LINC00662 promotes OC cell proliferation and tumorigenicity in vitro and in vivo

**Fig. 2:**
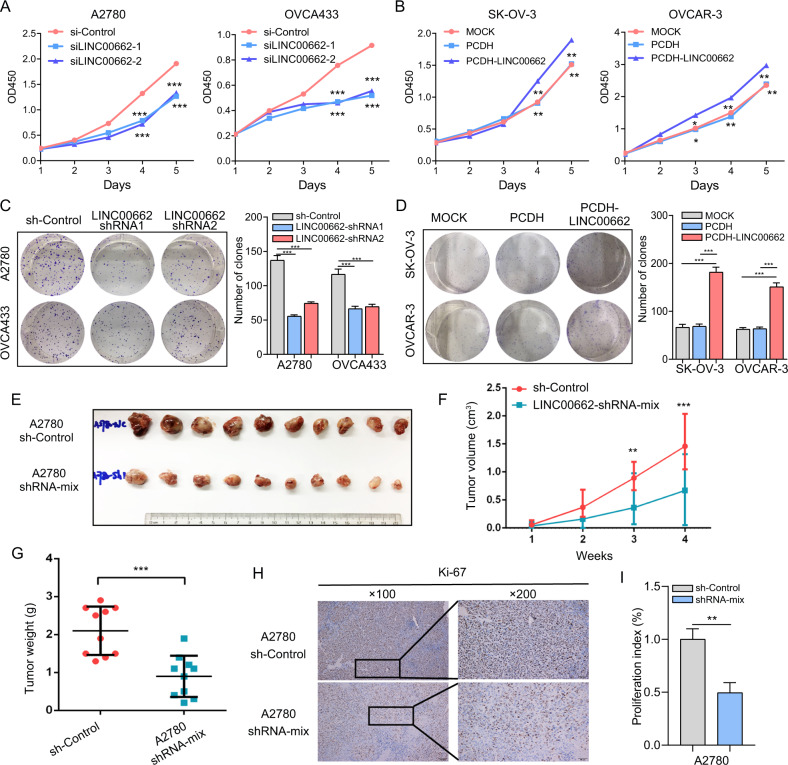
LINC00662 promotes OC cell proliferation in vitro and in vivo. A CCK-8 cell proliferation assays of A2780 and OVCA433 cells transfected with LINC00662 siRNAs or scrambled controls. **B** Cell proliferation assays of SK-OV-3 and OVCAR-3 cells infected with lentivirus expressing LINC00662 or control lentivirus. **C** Colony formation assay of A2780 and OVCA433 cells transfected with LINC00662 siRNAs or scrambled controls. **D** Colony formation assay of SK-OV-3 and OVCAR-3 cells infected with lentivirus expressing LINC00662 or control lentivirus. **E** Representative images of xenografts formed by subcutaneous injection of A2780 cells stably transfected with control or shRNA-mix into the right axilla of nude mice (*n* = 10 mice per group). **F**, **G** Growth curves and weights of tumors formed by stable A2780 cells infected with lentivirus for knockdown of LINC00662 or control lentivirus. **H**, **I** Immunohistochemical staining was conducted to compare proliferation indices via assessment of Ki‐67 expression. Data are shown as means ± SEMs. A two-tailed Student’s *t*-test was used for statistical analysis. *P < 0.05; **P < 0.01; ***P < 0.001. *P* < 0.05 was considered significant.

To further determine the oncogenic effects of LINC00662 on cell proliferation and tumorigenicity, we first measured the expression levels of LINC00662 in various OC cell lines (Supplementary Fig. S3A). Based on the high endogenous LINC00662 expression level in A2780 and OVCA433 cells, we identified two independent small interfering RNAs (siRNAs) to effectively reduce the LINC00662 RNA level (Supplementary Fig. S3B), whereas SK-OV-3 and OVCAR-3 cells, with low endogenous LINC00662 expression levels, were infected with lentivirus, harboring LINC00662 sequence within the PCDH vector to generate stable overexpression cell lines (Supplementary Fig. S3C). As indicated in the results, silencing of LINC00662 by specific siRNAs significantly inhibited A2780 and OVCA433 cell proliferation (Fig. 2A), whereas LINC00662 stable overexpression significantly decreased the proliferation ability of SK-OV-3 and OVCAR-3 cells (Fig. 2B). Consistent with these findings, repression and overexpression of LINC00662 significantly decreased (Fig. 2C) and promoted, respectively, the colony-forming ability of OC cells compared with the corresponding control cells (Fig. 2D). Furthermore, cell cycle distribution analysis showed that both silencing and overexpression of LINC00662 in OC cells significantly affected G1/S transition (Supplementary Fig. S3D, E). Similarly, western blot analysis demonstrated that knockdown of LINC00662 decreased cyclin E and p27 protein levels in A2780 and OVCA433 cells (Supplementary Fig. S3F), whereas overexpression of LINC00662 strongly increased cyclin E and p27 protein levels in SK-OV-3 and OVCAR-3 cells (Supplementary Fig. S3G).

To examine the effect of LINC00662 on tumorigenicity in vivo, we pooled two short-hairpin RNAs to establish cell lines with stable LINC00662 knockdown (Supplementary Fig. S3H), and cells expressing the resulting shRNA-mix were selected for use in a xenograft mouse model. Ten mice were injected subcutaneously with LINC00662-shRNA-mix and control cells, and all mice developed detectable tumors. The volumes of the tumors formed by the LINC00662 knockdown A2780 (LINC00662-shRNA-mix) cells were significantly smaller than those formed by the control cells (Fig. 2E, F). In addition, the average tumor weight was significantly lower in the LINC00662-shRNA-mix cell group than in the control group (Fig. 2G). Immunohistochemical analysis demonstrated that the Ki67 proliferation index was lower in tumors from LINC00662-shRNA-mix-transfected mice than in those from control mice. (Fig. 2H, I)

### LINC00662 drives OC cell invasion and metastasis in vitro and in vivo

**Fig. 3:**
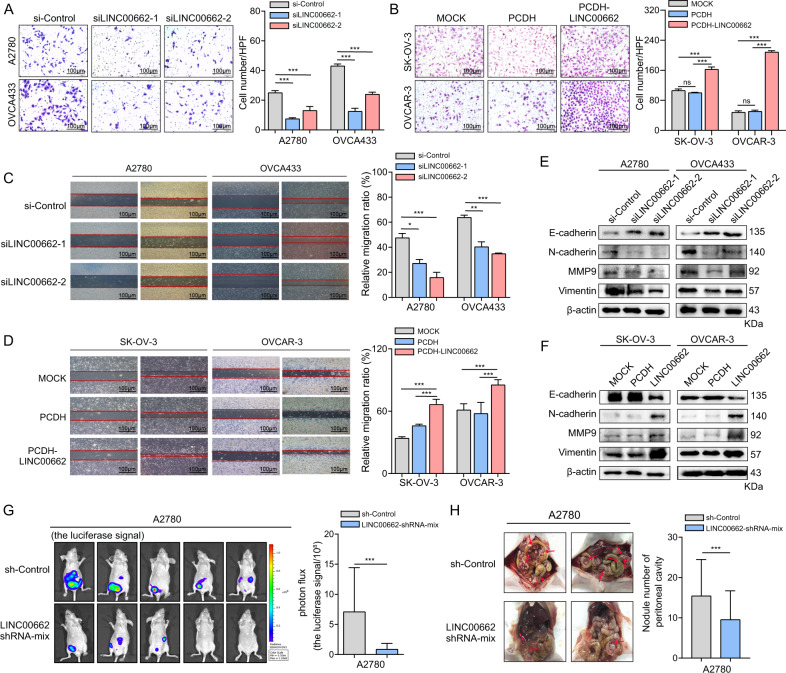
LINC00662 promotes OC invasion and metastasis in vitro and in vivo. **A** Transwell migration assays of A2780 and OVCA433 cells transfected with LINC00662 siRNAs or scrambled controls. The scale bar represents 50 μm. **B** Transwell migration assays of SK-OV-3 and OVCAR-3 cells infected with lentivirus expressing LINC00662 or control lentivirus. The scale bar represents 50 μm. **C**, **D** The migration ability of cells with LINC00662 knockdown or overexpression was assessed using wound-healing assays; images of A2780, OVCA433, SK-OV-3, and OVCAR-3 cells were acquired at 0 and 48 h post wounding. The scale bar represents 200 μm. **E**, **F** Western blotting results for N-cadherin, E-cadherin, MMP9, and Vimentin when repressing or overexpressing LINC00662 in A2780, OVCA433, SK-OV-3, and OVCAR-3 cells, respectively. **G** BALB/c nude mice were injected intraperitoneally with Luc-labeled A2780 cells with stable downregulation of LINC00662 or with control cells via the cavum abdominis; luciferase activities were assessed at 4 weeks posttransplantation (*n* = 5 mice per group). **H** Box plot showing the luciferase signals in the A2780 control and A2780 LINC00662-shRNA-mix groups. **I** Representative images of abdominal cavity metastases derived from two groups after sacrifice. **J** Quantification of the number of metastatic nodules of tumors in the abdominal cavities. Data are shown as means ± SEMs. A two-tailed Student’s *t*-test was used for statistical analysis. *P < 0.05; **P < 0.01; ***P < 0.001. *P* < 0.05 was considered significant. NS not significant.

The positive relationship between the level of LINC00662 RNA and the existence of distant metastasis prompted us to explore whether LINC00662 affects cell invasion and metastasis. The results of Transwell assays showed that siRNA-mediated LINC00662 knockdown suppressed the invasion of A2780 and OVCA433 cells (Fig. 3A) but that LINC00662 overexpression significantly enhanced SK-OV-3 and OVCA433 cells invasion (Fig. 3B). To further confirm the above results, we conducted additional Transwell assays with fluorescence staining, results indeed confirmed that knockdown of LINC00662 decreased the invasion of A2780 and OVCA433 cells (Supplementary Fig. S3I), whereas overexpression of LINC00662 enhanced the invasion of SK-OV-3 and OVCA433 cells (Supplementary Fig. S3J). Moreover, the wound-healing assays indicated that migration was suppressed when LINC00662 was silenced (Fig. 3C). Conversely, the promotive effect of LINC00662 on the migration ability of SK-OV-3 and OVCA433 cells was confirmed by LINC00662 overexpression (Fig. 3D). Accordingly, Consistent with these findings, the western blot analysis showed that downregulation of LINC00662 resulted in increased E-cadherin expression and decreased N-cadherin expression (Fig. 3E). The cell migration and invasion markers MMP2 and vimentin were also decreased when LINC00662 was downregulated (Fig. 3E). However, overexpression of LINC00662 led to increased N-cadherin, MMP9, and vimentin and decreased E-cadherin expression (Fig. 3F).

Next, we utilized an orthotopic model to examine peritoneal seeding ability of ovarian cells after 4 weeks post inoculation. A total of ten mice received an intraperitoneal injection of A2780 cells and were randomly divided into control and test groups (five mice per group). The extent of peritoneal seeding of A2780 cells was examined by evaluating the expression of luciferase, which is considered a tracer molecule for in vivo imaging of tumor status (Fig. 3G). We observed a significant reduction in the luciferase signal in the A2780 shRNA-mix group compared to the control group (Fig. 3H). The mice were then sacrificed, and LINC00662-silenced A2780 cells substantially abrogated the capacity of the cells to form secondary tumors in abdominal cavity (Fig. 3I, J).

### LINC00662 interacts with HNRNPH1 and GRP78 in OC cells

**Fig. 4:**
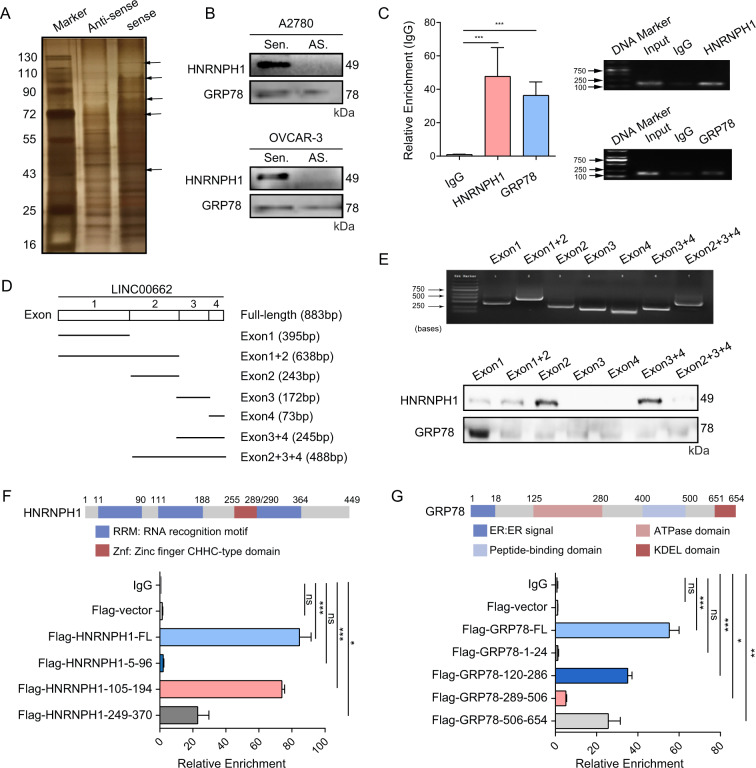
LINC00662 physically interacts with HNRNPH1 and GRP78 in OC cells. **A** LINC00662-sense and LINC00662-antisense probes were biotinylated, transcribed in vitro, and incubated with whole-cell lysates of A2780 cells for RNA pulldown assays. After silver staining, the 40-130-kDa LINC00662 sense-specific bands (arrows), which repeatedly appeared in two independent assays, were excised and analyzed by mass spectrometry. **B** Immunoblot analysis of specific associations of HNRNPH1 or GRP78 with LINC00662 from two RNA pulldown assays in different OC cell lines. **C** RIP assays using anti-HNRNPH1 and anti-GRP78 antibodies showed that HNRNPH1 and GRP78 specifically interact with LINC00662 in A2780 cells. The qRT-PCR results for the RIP assay precipitates are shown on the left. The results of agarose gel electrophoresis of the PCR products are shown on the right. **D** Schematic of the LINC00662 truncations used for pulldown assays (exon 1, 395 bp; exon 1 + 2, 638 bp; exon 2, 243 bp; exon 3, 172 bp; exon 4, 73 bp; exon 3 + 4, 245 bp; exon 2 + 3 + 4, 488 bp). **E** Immunoblot analysis of HNRNPH1 and GRP78 in samples precipitated by biotinylated LINC00662 truncations. Deletion mapping to identify the domains of HNRNPH1 (**F**) or GRP78 (**G**) that bind to LINC00662. RIP analysis for LINC00662 enrichment in cells transiently transfected with the full-length or truncated FLAG-tagged constructs. Data are shown as means ± SEMs. A two-tailed Student’s *t*-test was used for statistical analysis. *P < 0.05; **P < 0.01; ***P < 0.001. *P* < 0.05 was considered significant. NS not significant.

To elucidate the molecular mechanism by which LINC00662 promotes oncogenic behaviors in OC cells, we next performed pulldown assays and analyzed the precipitated proteins by mass spectrometry (MS) to search for potential LINC00662-interacting proteins. In two independent LINC00662 pulldown experiments, many different specific bands between 40 and 130 kDa were enriched (Fig. 4A). MS analysis revealed 32 proteins based on the number of unique peptides (>5) and the number of repeated peptides (>8) (Supplementary Table S2). Immunoblot analysis of different OC cell lines confirmed that two of the candidates, HNRNPH1 and GRP78, interact directly with LINC00662 (Fig. 4B). Moreover, in RIP assays, LINC00662 was markedly enriched when antibodies against HNRNPH1 and GRP78 were used, as determined by qPCR analysis of coprecipitated RNAs (Fig. 4C, left) and RT-PCR with agarose gel electrophoresis (Fig. 4C, right). Moreover, a series of truncation mutants were constructed based on the four exons that compose LINC00662 (Fig. 4D). The “Exon 1 + 2”, “Exon 2”, “Exon 3 + 4”, and “Exon 2 + 3 + 4” fragments of LINC00662 may predominantly mediate the interaction of LINC00662 with HNRNPH1, the “Exon 1” and “Exon 1 + 2” fragments is necessary and sufficient for its association with GRP78 (Fig. 4E). Next, the results of RIP assays of FLAG-tagged full-length and truncated HNRNPH1 and GRP78 fragments showed that the RNA recognition motif (RRM, aa 111-188) of HNRNPH1 and the ATPase domain (aa 125-280) of GRP78 physically associate with LINC00662 (Fig. 4F, G). In addition, the KDEL domain (aa 651-654) of GRP78 may be responsible for the interaction of GRP78 with LINC00662 in OC cells (Fig. 4G). Collectively, these results indicate that LINC00662 binds specifically with both HNRNPH1 and GRP78.

### HNRNPH1-stabilized LINC00662 inhibits GRP78 ubiquitination and degradation

**Fig. 5:**
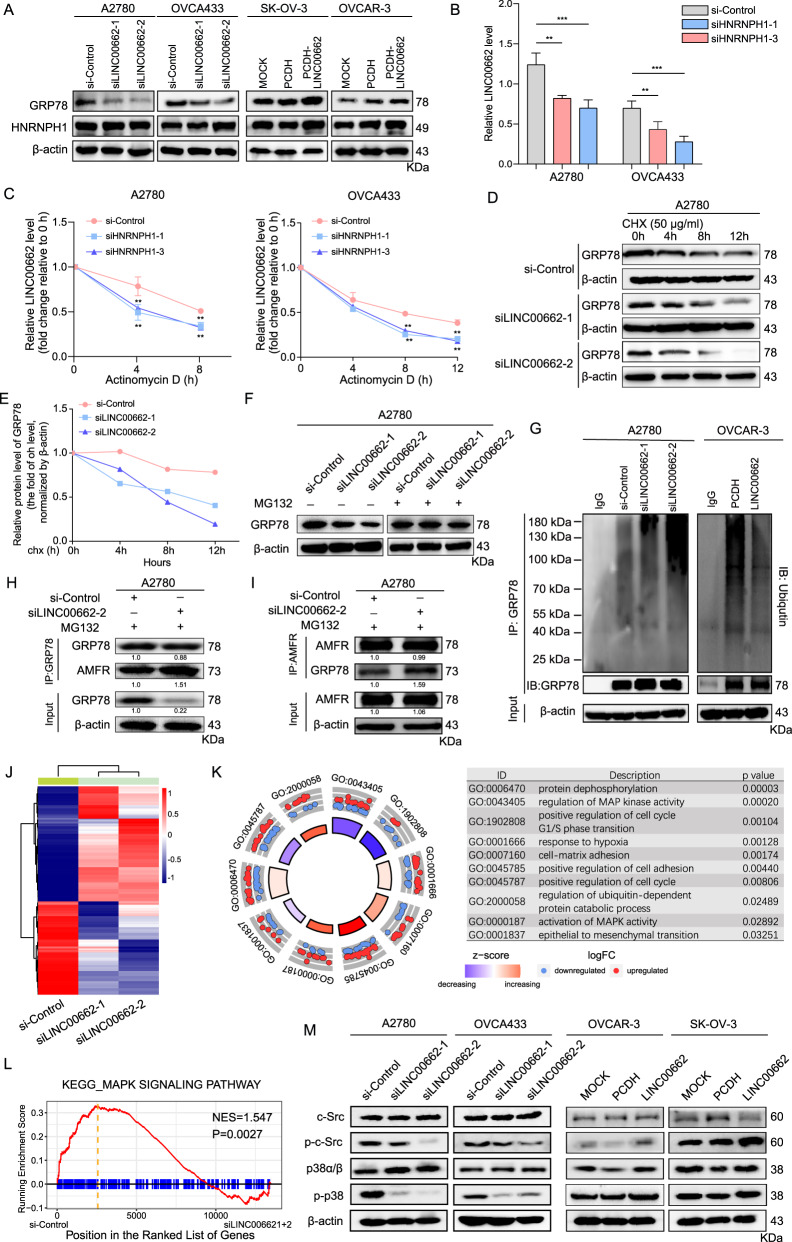
HNRNPH1-stabilized LINC00662 blocks ubiquitin-proteasome-dependent GRP78 degradation. **A** Immunoblot analysis of the protein levels of HNRNPH1 and GRP78 after LINC00662 activation or knockout. β-Actin was used as the internal control. **B** Relative LINC00662 levels when silencing HNRNPH1 with specific siRNAs in A2780 and OVCA433 cells. **C** Half-life of LINC00662 in A2780 and OVCAR-3 cells with HNRNPH1 knockdown after treatment with 2.5 μM actinomycin D for the indicated times. **D**, **E** A2780 cells transfected with LINC00662 siRNAs were treated with CHX (50 mg/ml) for the indicated times. **D** Immunoblot analysis of GRP78 levels in whole-cell extracts. **E** Densitometric analysis of the GRP78 protein bands, whereby the relative fold change in the level is with respect to the level at 0 h. **F** A2780 cells transfected with LINC00662 siRNAs were treated with MG132 (25 mM) for 12 h; immunoblot analysis of GRP78 levels in the indicated cells. **G** Lysates from A2780 cells with LINC00662 knockdown or OVCAR-3 cells with LINC00662 overexpression were immunoprecipitated (IP) with either control IgG or an anti-GRP78 antibody and then immunoblotted to assess ubiquitin and GRP78 levels. **H**, **I** Immunoprecipitation to detect the association between GRP78 and AMFR after LINC00662 knockdown. In brief, lysates of A2780 cells transfected with LINC00662 siRNA or scrambled control siRNA and treated with 25 mM MG132 for 4 h were subjected to coimmunoprecipitation with antibodies specific for GRP78 and AMFR followed by western blot analysis using the indicated antibodies. **J** Heatmap of DEGs identified by RNA-seq. **K** GO enrichment analysis of DEGs. **L** MAPK signaling pathway enrichment in A2780 cells transfected with LINC00662 siRNA or scrambled control siRNA, as shown by GSEA. **M** Immunoblot analysis of c-Src (Tyr419/Tyr424) and p38 (Tyr182) phosphorylation in A2780 and OVCA433 cells transfected with LINC00662 siRNAs and in OVCAR-3 and SK-OV-3 cells with transient overexpression of LINC00662. Data are shown as means ± SEMs. A two-tailed Student’s *t*-test was used for statistical analysis. **P < 0.01; ***P < 0.001. *P* < 0.05 was considered significant.

Given that LINC00662 interacts with HNRNPH1 and GRP78 in OC cells, we characterized the molecular consequences of these interactions. Interestingly, LINC00662 did not affect either the protein or mRNA level of HNRNPH1 (Fig. 5A, Supplementary Fig. S4A). Although LINC00662 did not significantly affect the mRNA level of GRP78 (Supplementary Fig. S4B), the protein level of GRP78 was dramatically reduced when LINC00662 was silenced and increased when LINC00662 was overexpressed (Fig. 5A). Given that LINC00662 modulation did not affect either the protein or mRNA level of HNRNPH1, we first designed three siRNAs targeting HNRNPH1 (Supplementary Fig. S4C), and found that silencing HNRNPH1 significantly reduced the level of LINC00662 in A2780 and OVCA433 cells (Fig. 5B). Bioinformatic analysis using the GEPIA database (http://gepia.cancer-pku.cn/index.html) revealed that the HNRNPH1 level was positively correlated with the LINC00662 level (*r* = 0.39, *P* < 0.001, Supplementary Fig. S4D). Based on the abovementioned results and considering that HNRNPH1 belongs to the RNA-binding protein (RBP) family, we attempted to evaluate whether LINC00662 can be regulated by HNRNPH1. We used actinomycin D, which effectively inhibits de novo RNA synthesis, to evaluate the stability of LINC00662. Knockdown of HNRNPH1 resulted in a decreased half-life and expression level of LINC00662 (Fig. 5C) in A2780 and OVCA433 cells, suggesting a role for HNRNPH1 in regulating the stability of LINC00662.

Considering the effect of LINC00662 on the GRP78 protein level, we treated A2780 and OVCAR-3 cells with the protein synthesis inhibitor cycloheximide and found that the protein stability and half-life of GRP78 were dramatically decreased after LINC00662 silencing in A2780 and OVCAR-3 cells (Fig. 5D, E, Supplementary Fig. S4E). Furthermore, GRP78 protein degradation in A2780 cells was accelerated by transfection of LINC00662 siRNAs and inhibited by treatment with the proteasome inhibitor MG132 (Fig. 5F), suggesting that LINC00662 prevents proteasome-dependent degradation of GRP78 in OC cells. As expected, the ubiquitination level of GRP78 was significantly increased in LINC00662 siRNA-treated cells but was decreased in LINC00662-overexpressing cells (Fig. 5G). A recent report identifying AMFR as an E3 ligase for GRP78 ubiquitination and degradation [13] prompted us to investigate the function of LINC00662 in the interaction between the E3 ligase AMFR and GRP78 in OC cells. Indeed, LINC00662 knockdown enhanced the interaction between AMFR and GRP78 (Fig. 5H, I). Collectively, our data demonstrate that LINC00662 may be stabilized by HNRNPH1 and promote proteasome-mediated ubiquitination and degradation of GRP78.

### LINC00662 regulates the p38/MAPK signaling pathway

To better understand the molecular mechanisms by which LINC00662 enhances the malignant behaviors of OC cells, we performed RNA sequencing (RNA-seq) analysis to obtain the transcriptional profiles of A2780 cells, with LINC00662 knockdown by two siRNAs (Fig. 5J, Supplementary Table S3). We next randomly selected a few top differentially expressed genes to validate the accuracy of this RNA-seq dataset by RT-PCR (Supplementary Fig. S5A, B). Gene ontology analysis showed that LINC00662 regulated multiple biological processes in OC cells, especially processes related to MAPK activity, cell cycle arrest, and epithelial to mesenchymal transition (Fig. 5K), which confirmed our previous results. Moreover, the MAPK pathway was significantly enriched in gene set enrichment analysis plots; indeed, it was the top-ranked signaling pathway (Fig. 5L, Supplementary Table S4). We then performed immunoblotting to examine the effects of LINC00662 on MAPK signaling. Considering that GRP78 interacts with LINC00662 and it is reported to frequently be overexpressed on the surface of OC cells, where it contributes to activating the c-Src and p38/MAPK signaling pathways [14]. We sought to determine whether LINC00662 may influence p38/MAPK signaling pathways through c-Src. LINC00662 knockdown by specific siRNAs inhibited the phosphorylation of p38 (Tyr182) and c-Src (Tyr419/Tyr424) in A2780 and OVCA433 cells, whereas LINC00662 overexpression significantly promoted the phosphorylation of these sites in OVCAR-3 and SK-OV-3 cells (Fig. 5M). In contrast, the total protein levels of p38α/β and c-Src were not significantly altered. To further establish the correlation between LINC00662 and p38/MAPK signaling, we also evaluated the levels of p-p38 in patients in cohort 2 (Supplementary Fig. S6A). Immunohistochemical analysis showed that strong p-p38 staining was consistently identified in the high LINC00662 expression group (Supplementary Fig. S6B). Collectively, these results demonstrate that LINC00662 activates the p38/MAPK signaling pathway.

### GRP78 is a functional mediator of LINC00662 in OC cells

**Fig. 6:**
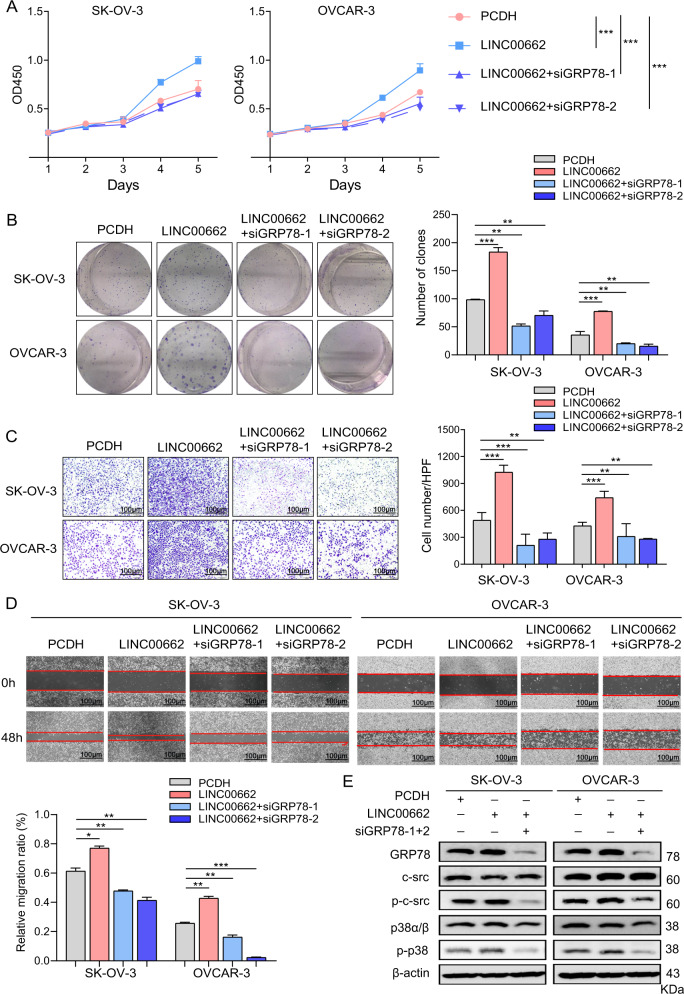
The oncogenic effects of LINC00662 are mediated via GRP78/p38 signaling. **A** The increase in the viability of LINC00662-overexpressing OC cells was abolished by GRP78 knockdown. Cell viability was measured by CCK-8 assays. **B** Colony formation rescue assays were performed after GRP78 silencing in PCDH-LINC00662 cells. **C**, **D** Transwell migration and wound-healing rescue assays were performed after LINC00662 silencing in OVCAR-3 and SK-OV-3 cells with transient silencing of GRP78. **E** Immunoblot analysis of c-Src (Tyr419/Tyr424) and p38 (Tyr182) phosphorylation rescue after LINC00662 overexpression in OVCAR-3 and SK-OV-3 cells with transient silencing of GRP78. Values shown are means ± SEMs, *n* = 3 in **A**–**D**. β-Actin was used as the internal control in **E**. Data are shown as means ± SEMs. A two-tailed Student’s *t*-test was used for statistical analysis. *P < 0.05; **P < 0.01; ***P < 0.001. *P* < 0.05 was considered significant.

Considering the effect of LINC00662 on GRP78 protein stability, we hypothesized that LINC00662 may exert its biological functions through GRP78 in OC cells. To investigate this hypothesis, we first investigated the roles of GRP78 in OC cells. Knockdown of GRP78 by specific siRNAs was validated by western blot analysis (Supplementary Fig. S7A). Moreover, transfection with GRP78-specific siRNAs markedly reduced the proliferation, colony formation, invasion and migration of A2780 and OVCAR-3 cells (Supplementary Fig. S7B–E), demonstrating the important role of GRP78 in OC cells. In addition, to elucidate whether LINC00662 functions in OC cells in a GRP78-mediated manner, we performed Cell Counting Kit-8 (CCK-8) and colony formation assays. GRP78 knockdown markedly impaired the promotive effect of LINC00662 overexpression on cell proliferation (Fig. 6A, B). Similarly, the effect of LINC00662 on the invasion and migration of SK-OV-3 and OVCAR-3 cells was partially attenuated by the repression of GRP78 (Fig. 6C, D). As GRP78 is the functional downstream target gene of LINC00662, we determined that GRP78 contributes to the effect of LINC00662 on p38/MAPK signaling in OC cells. Indeed, GRP78 knockdown rescued the oncogenic effects of LINC00662 overexpression on p38/MAPK signaling in OVCAR-3 and SK-OV-3 cells (Fig. 6E). Collectively, these results show that LINC00662 activates the p38/MAPK signaling pathway by interacting with GRP78.

### The LINC00662/GRP78 axis is a potential target and correlates with patient outcomes

**Fig. 7:**
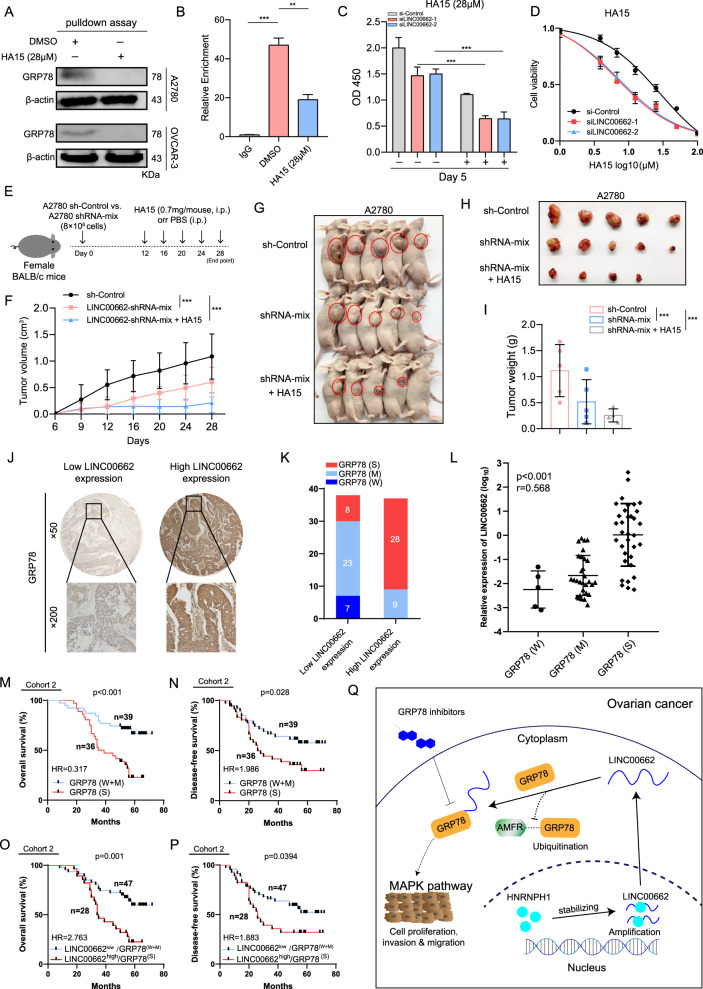
Illustration of the LINC00662/GRP78 axis in OC. **A** RNA pulldown assays to determine the specific association of GRP78 and LINC00662 in cells treated with the GRP78 inhibitor HA15 at the indicated concentration for 48 h. **B** RIP assays were performed using anti-GRP78 antibodies in cells treated with the GRP78 inhibitor HA15 at the indicated concentration for 48 h. **C** CCK-8 assays of cells with LINC00662 silencing after treatment with the GRP78 inhibitor HA15 at the indicated concentration for 5 days. **D** IC_50_ values were determined in cells transfected with LINC00662 siRNAs and exposed to the GRP78 inhibitor for 4 days. **E** Summary of experimental design to study the effect of LINC00662 knockdown in combination with HA15 in ovarian cancer is shown. **F** Tumor volume of mice was measured on indicated days. **G**, **H** Images of the excised tumors from the indicated mice at 28 days after injection (five mice per group). **I** Average weight of excised tumors from the indicated mice (*n* = 5). Each bar represents the median values ± quartile values. **J** Representative images of immunohistochemical staining for GRP78 in tumor tissues from patients with OC (*n* = 75) with low or high levels of LINC00662. **K** Percentages of specimens showing different levels of GRP78 staining in the low or high LINC00662 expression groups (*n* = 75; W, weak; M, moderate; S, strong). **L** Direct correlation between LINC00662 and GRP78 protein expression, as assessed using Spearman correlation analysis (*n* = 75, chi-square test). Expression of LINC00662 and the immunohistochemical score for the GRP78 protein in each OC specimen were examined separately. **M**, **N** Kaplan–Meier analysis of the OS and DFS of OC patients stratified according to the GRP78 expression level (log-rank test). **O**, **P** Kaplan–Meier analysis of the OS and DFS of OC patients stratified according to the combination of LINC00662 expression and GRP78 protein level (log-rank test). **Q** Proposed working model of this study. The LINC00662 locus is amplified, and its transcript is stabilized by HNRNPH1 in OC; LINC00662 exerts its oncogenic effects by interacting with and stabilizing GRP78, thereby activating the MAPK signaling pathway. Values shown are means ± SEMs. A two-tailed Student’s *t*-test was used for statistical analysis. **P < 0.01; ***P < 0.001. *P* < 0.05 was considered significant. Data are shown as means ± SEMs.

A recent study identified a specific small molecule inhibitor of GRP78, termed HA15 [15]. HA15 binds to GRP78 and inhibits its ATPase activity, which was demonstrated to be responsible for its interaction with LINC00662, suggesting the clinical application of this inhibitor in OC. Thus, we evaluated whether HA15 can be used to target the LINC00662/GRP78 axis in OC cells. The results of RNA pulldown assays showed that LINC00662 did not interact with GRP78 in A2780 and OVCAR-3 cells after treatment with HA15 (Fig. 7A). The results of RIP assays in A2780 cells confirmed that HA15 interfered with the interaction between LINC00662 and GRP78 (Fig. 7B). Moreover, in OC cells with LINC00662 silencing, treatment with HA15 led to a sharp reduction in proliferation (Fig. 7C). Notably, the IC_50_ of HA15 (28 μM) in LINC00662-silenced cells was dramatically decreased compared with that in control cells (Fig. 7D). To further explore the functional significance of the LINC00662/GRP78 axis in its protumor role in OC, we performed xenograft studies by subcutaneously injecting A2780 sh-Control and A2780 LINC00662-shRNA cells into female BALB/c mice. After 12 days, the mice were treated intraperitoneally with HA15 (0.7 mg/mouse) or PBS. On day 28, the mice were sacrificed for further analysis (Fig. 7E). Tumors formed by LINC00662-shRNA cells grew at a much slower rate than tumors formed by sh-Control cells, consistent with the results of previous in vivo experiments (Fig. 2E–G). Moreover, Silencing LINC00662 resulted in a profound decrease in tumor volume and tumor weight in the HA15-treated LINC00662-shRNA group (Fig. 7F–I). These findings reveal that the LINC00662/GRP78 axis is a potential therapeutic target in OC.

To further establish the correlation between LINC00662 and GRP78 in OC tissues, we used immunohistochemistry to evaluate the GRP78 protein level in cohort 2, which was used for determination of the LINC00662 RNA level and contained 75 OC samples. Staining of GRP78 was categorized as strong (S), medium (M) or weak (W), and strong GRP78 staining was more common in the high LINC00662 expression group than in the low LINC00662 expression group (Fig. 7J, K). Furthermore, in OC tissues, GRP78 protein levels correlated positively with LINC00662 levels (*r* = 0.568, *P* < 0.001, Fig. 7L), confirming the positive regulation of GRP78 by LINC00662. Considering the low number of patients with weak staining for GRP78 (*n* = 7), we combined the weak and medium staining groups as one for subsequent analysis. Kaplan–Meier survival curves for 75 OC patients revealed that patients with strong GRP78 staining had lower OS rates (*P* < 0.001, Fig. 7M) and DFS (*P* = 0.028, Fig. 7N) than those with medium or weak GRP78 staining. Notably, patients with both high LINC00662 expression and strong GRP78 staining showed significantly reduced OS (*P* = 0.001, Fig. 7O) and DFS (*P* = 0.0394, Fig. 7P). These results indicate that GRP78 staining correlates positively with LINC00662 expression and predicts poor clinical outcomes in patients.

## Discussion

Chromosomal CNAs are often observed in many types of cancer and typically lead to the activation of oncogenes or inactivation of tumor suppressors [16]. For instance, CCNE1 amplification has been identified as a primary oncogenic driver in a subset of HGSOCs, and it is a potential predictive marker for resistance to cytotoxic chemotherapies [17–19]. Although many studies have addressed the functions and mechanisms of CNAs in OC, little is known about functional noncoding sequences, especially lincRNAs. In this study, we identified three lincRNAs with the top-ranked correlations between their CNA and expression levels. Among these lincRNAs, LINC00674 has been reported to promote tumor progression in hepatocellular carcinoma [20], colon cancer [21], and glioblastoma [22], whereas the function of LINC00493 is largely unknown. LINC00662, which is expressed from a region on chromosome 19q11, showed the strongest correlation between its CNA levels and RNA levels in OC. The LINC00662 locus was found to be amplified in OC tissues and to have oncogenic properties driving the progression of OC. Importantly, LINC00662 expression correlated positively with FIGO stage, and distant metastasis. In addition, patients with higher expression of LINC00662 were predicted to have poorer OS outcomes, suggesting that LINC00662 expression is a promising prognostic marker in OC. In addition, recent reports confirmed the prognostic role of LINC00662 in multiple types of cancer [23–27]. These findings suggest that LINC00662 may be an oncogene in many cancers in addition to OC.

Accumulating evidence has shown that lncRNAs perform global cellular functions by binding with proteins, miRNAs, or other cellular factors [28]. LINC00662 can trigger malignant progression as a competing endogenous RNA by sponging different miRNAs [23, 24, 27] or altering genomic methylation profiles by binding to the mRNA of MAT1A and the ACHY protein in hepatocellular carcinoma [29]. In the present study, we first identified HNRNPH1 and GRP78 as *bona fide* interacting partners of LINC00662 and demonstrated the oncogenic function of the HNRNPH1-LINC00662-GRP78 axis in OC cells. HNRNPH1 binds primarily to the “Exon 1 + 2”, “Exon 2”, “Exon 3 + 4”, and “Exon 2 + 3 + 4” fragments of LINC00662. However, the “Exon 1” and “Exon 1 + 2” fragment is responsible for the interaction of LINC00662 with GRP78. The main binding fragments of LINC00662 between HNRNPH1 and GRP78 are different, so it is reasonable to speculate that these three molecules do not bind to each other and may exert their biological functions via different mechanisms. HNRNPH1, belonging to the heterogeneous nuclear ribonucleoprotein family, is an RBP that is involved in premRNA splicing and mRNA trafficking and stability [30, 31]. Specifically, HNRNPH1 regulates the splicing of the proapoptotic effector BCL-X and maintains 3′-end processing of the p53 premRNA [32, 33]. Splicing of EWS-FLI1 in Ewing sarcoma has been shown to be regulated by HNRNPH1 [34], and HNRNPH1 plays a primarily oncogenic role in the nucleus [35]. However, other evidence indicates that HNRNPH1 can localize to the cytoplasm in cancer cells [36], suggesting the possibility of interaction between HNRNPH1 and LINC00662. Considering that cytoplasmic RBPs are primarily involved in stabilizing mRNAs or in mediating their translation [37], our study demonstrated that HNRNPH1 interacts with LINC00662 and strongly impacts its stability. This result enhances the understanding of LINC00662 expression regulation, which results from its genomic alterations (copy number amplification) and posttranscriptional level (stabilization by HNRNPH1) in OC cells.

Moreover, we identified GRP78 as a downstream effector of LINC00662. The 78-kDa glucose-regulated protein GRP78, also called BiP and HSP5A, is a multifunctional protein with activities far outside its well-known role in the unfolded protein response, which is activated in cells under ER stress [14]. As an oncogene, GRP78 influences tumor cell survival, proliferation, and chemoresistance development in multiple types of cancer, including OC [38], breast cancer [39], prostate cancer [40], and colorectal cancer [41]. In this study, we found LINC00662 to be associated with GRP78 and to promote its degradation via the ubiquitin-proteasome pathway in OC cells. More importantly, we showed that LINC00662 inhibits the E3 ubiquitin ligase AMFR from binding to and stabilizing GRP78, consistent with previous reports about the ubiquitination of GRP78 [13]. The newly discovered functions of GRP78 are dependent on its intracellular localization. In cells, GRP78 is found in other compartments in addition to the ER, such as the cell surface [14], suggesting its role in signal transduction. Given that LINC00662 stabilizes the GRP78 protein, we hypothesized that such interaction might influence the downstream signaling in OC cells. Furthermore, LINC00662 expression and GRP78 protein staining were found to be independent prognostic factors in OC.

In addition, our gene expression profiling analysis in OC cells provided evidence that LINC00662 potentially regulates the MAPK signaling pathway. GRP78 likely promotes tumor cell invasion by activating Src/MAPK pathways in lung tumors [42]. Cell-surface GRP78 can bind to Src [43], and several lines of evidence suggest that the Src protein can activate the p38 MAPK pathway [44, 45]. Considering that p38/MAPK regulates a wide range of cellular processes, including cell metabolism, proliferation, motility, apoptosis, survival, and differentiation [46], it is not surprising that LINC00662 can activate the GRP78/p38 pathway in OC cells, thus promoting the progression of OC.

Clinically, the GRP78 inhibitor HA15 specifically inhibits the activity of GRP78 in malignant pleural mesothelioma and melanoma by binding to its ATPase domain [15, 47], which is the domain responsible for the interaction of GRP78 with LINC00662. HA15 abolished the association between LINC00662 and GRP78 by competitively binding to the ATPase domain of GRP78, which resulted in an additive inhibitory effect on OC cell proliferation in combination with LINC00662 knockdown. Our results strongly indicate that the newly identified LINC00662/GRP78 axis is a potential therapeutic target in OC and that specific small molecule inhibitors of GRP78 can be used to treat OC patients with high expression levels of LINC00662.

In summary, we found that the lincRNA LINC00662, located on chromosome 19q11, is amplified, overexpressed, and stabilized by HNRNPH1 in OC cells. LINC00662 can physically interact with GRP78, resulting in stabilization of GRP78 and progression of OC cells. These results provide the first evidence of a LINC00662/GRP78/p38 axis in OC (Fig. 7Q). The discovery of LINC00662 provides mechanistic insight into its oncogenic roles and pivotal effects as a biomarker and therapeutic target.

## Materials and methods

### Patient series

Two sets of human OC samples were used in this study. For Cohort 1, 40 OC specimens and 36 normal ovarian tissues were collected as DNA samples. For Cohort 2, 75 unpaired OC and normal ovarian tissues from patients were obtained from the surgical specimen archives. OC tissues were collected from participants at primary debulking surgery, and normal ovarian tissues were obtained from the normal ovaries of donors during surgery for other gynecological diseases without ovarian involvement at FUSCC. A summary of the clinical information for cohort 2 is available online in Supplementary Table S5. In addition, a tissue microarray was constructed using specimens from cohort 2. All human materials were obtained with informed consent, and the study was approved by the Ethical Review Committee of the World Health Organization of the Collaborating Center for Research in Human Production and authorized by the Human Ethics Committee of FUSCC.

### RNA isolation, reverse transcription and qRT-PCR

Total RNA was extracted from tissue samples of Cohort 2 and cell lines using TRIzol reagent (Invitrogen, Carlsbad, CA, USA) according to the manufacturer’s protocol. First-strand cDNA was synthesized using a PrimeScript™ Reverse Transcriptase Kit (Takara, Dalian, China). Relative RNA levels were measured by qRT-PCR with a LightCycler 480 PCR System (Roche, USA) using the SYBR Green (Takara) method. The primer sequences are listed in Supplementary Table S6. The relative quantification value for each target gene is expressed as the 2^−ΔΔCT^ value. β-Actin was employed as the internal reference for mRNA expression.

### Western blot analysis

Proteins were subjected to sodium dodecyl sulfate-polyacrylamide gel electrophoresis and transferred to nitrocellulose membranes (GE, CT, USA). After blocking with nonfat milk, the membranes were incubated first with primary antibodies and then with secondary antibodies. The antibodies used to obtain the data shown in the main figures are provided in Supplementary Table S7.

### Statistical analysis

All statistical analyses were performed using SPSS 18.0 (IBM, SPSS, Chicago, IL, USA). The significance of differences between groups was estimated using Student’s *t*-test, the chi-square (*χ*^2^) test, or the Wilcoxon test, as appropriate. OS and DFS rates were calculated using the Kaplan–Meier method and compared with the log-rank test. The risk factors for patients’ prognosis were evaluated using univariate and multivariate Cox regression analyses. All statistical analyses were performed using two-tailed *P* values, and *P* < 0.05 was considered statistically significant (*P < 0.05, **P < 0.01, and ***P < 0.001). Figures were generated with GraphPad Prism 8 and Adobe Illustrator software. Unless stated otherwise, the data are presented as the mean ± SEM values from one representative experiment of three independent experiments, and every representative experiment was performed with experimental triplicates.

## Supplementary information

Supplementary Information

Supplementary Table S1

Supplementary Table S2

Supplementary Table S3

Supplementary Table S4

Supplementary Table S5

Supplementary Table S6

Supplementary Table S7
