# Supplementary material for: HNRNPH1-stabilized LINC00662 promotes ovarian cancer progression by activating the GRP78/p38 pathway
Source: Oncogene. 2021 Jun 19;40(29):4770–82. doi: 10.1038/s41388-021-01884-5 (PMC8298204; doi:10.1038/s41388-021-01884-5)
Supplement: Supplementary file 1 — Supplementary Information [file 41388_2021_1884_MOESM1_ESM.docx]

**Supplementary Information**

**Supplementary Materials and Methods**

**Genomic DNA copy number alteration analysis of lincRNAs**

Copy number alteration (CNA) dataset of 358 OC patients from The Cancer Genome Atlas (TCGA) which were retrieved from the GDC data portal (https://portal.gdc.cancer.gov/). The CNAs in 294 LINC-prefixed lincRNAs were identified by annotating lincRNAs in the reference genome file for which lincRNA information was available. The edited copy number profiles of 358 OC patients were reanalyzed using the GISTIC algorithm. Potential driver lincRNAs were selected with the criteria as follows: (1) lincRNAs with an absolute value of the cutoff for copy number amplification or deletion of greater than 0.3; (2) the CNA was present in at least three tumor samples; (3) the ranking of the lincRNA among the 294 lincRNAs according to the correlation between their CNAs and RNA expression levels was considered.

Genomic DNA was extracted from tissues using the TIANamp Genomic DNA Kit (TIANGEN, Beijing, China) following the manufacturer’s guidelines. Genomic copy number was measured by quantitative real-time polymerase chain reaction (qRT-PCR) assays using SYBR Green (Takara, Japan) with a LightCycler 480 PCR System (Roche, USA) in cohort 1 as described previously [1]. The number of copies of the target genes in each test sample was determined by relative quantitation (RQ) using the comparative CT (2−ΔΔCT) method.

**Cell lines, culture conditions and treatments**

TOV21G, OVCAR-3, CAOV-3 and SK-OV-3 cells were obtained from American Type Culture Collection (ATCC, Manassas, Virginia, USA). A2780, OVCA429, OVCAR433, OVCAR-4 and ES-2 cells were purchased from the Cell Resource Center, Peking Union Medical College, which is the headquarters of the National Infrastructure of Cell Line Resource (NSTI, Beijing, China). All cell lines were maintained at 37°C in 5% CO_2_ and cultured in Dulbecco’s modified Eagle's medium supplemented with 10% fetal bovine serum (FBS), 2 mM L-glutamine, 1 mM sodium pyruvate, and 100 U/ml penicillin-streptomycin (all obtained from Gibco-BRL, Grand Island, NY, USA). Cell lines received in 2016 were tested for authenticity in 2018 using short tandem repeat (STR) genotyping.

For cell treatment, cells were incubated with 50 μg/ml cycloheximide (CHX, Sigma-Aldrich) for the indicated durations. Cells were treated with the proteasome inhibitor MG132 (5 μM, Selleckchem) for 6 h. The small molecule GRP78 inhibitor HA15 was purchased from MCE (Shanghai, China).

**5′ and 3′ RACE analyses**

To obtain the full-length sequence of LINC00662, we performed 5'-RACE and 3'-RACE analyses using a SMARTer™ RACE cDNA Amplification Kit (Clontech, Palo Alto, CA, USA) according to the manufacturer’s instructions. The sequences of the gene-specific PCR primers used for the RACE analyses are provided in Supplementary Table S6.

**Subcellular fractionation analysis**

Cytoplasmic and nuclear fractions of A2780 and SK-OV-3 cells were collected according to the Nuclear/Cytoplasmic Isolation Kit instructions (Thermo Fisher Scientific, Carlsbad, California, USA). β-Actin was used as the endogenous control for cytoplasmic expression and U6 small nuclear RNA as that for nuclear expression.

**Lentiviruses, siRNAs, and transfection**

The sequences of the siRNA oligonucleotides targeting LINC00662, HNRNPH1 and GRP78 and the negative control siRNA are provided in Supplementary Table S6. All constructs were purchased from Synbio Technologies (Suzhou, China). siRNA transfection was performed with RNAiMAX (Invitrogen) according to the manufacturer’s instructions. The human LINC00662 sequence was amplified from A2780 cell cDNA and cloned into the BamHI and EcoRI sites of the lentiviral expression vector PCDH to generate PCDH-LINC00662. Additionally, plasmids containing full-length and truncated fragments of human Flag-tagged HNRNPH1 and GRP78, as designed based on GenBank accession numbers NM_005520.2 and NM_005347.4 respectively, were purchased from LncBIO (Shanghai, China). HEK293T cells were transfected with PCDH-LINC00662 along with the packaging and envelope plasmids psPAX2 and pMD2.G, respectively, using Lipofectamine 2000 (Invitrogen) according to the manufacturer’s instructions. The virus particles were collected 48 h after transfection. OC cells were infected with lentivirus transducing units using 1 μg/ml polybrene (Solarbio, Beijing, China).

**Cell proliferation and colony formation assays**

Cells were seeded in 96-well flat-bottom plates, with each well containing 2000 cells in 100 μl. After culture for the indicated duration, cell viability was assessed using CCK-8 assays (Dojindo, Kumamoto Prefecture, Japan). Each experiment was performed with experimental triplicates and repeated three times, with measurement for 5 consecutive days. For colony formation assays, 1000 cells were seeded in 6-well plates and incubated in the corresponding medium supplemented with 10% FBS at 37°C. After two weeks, the cells were fixed and stained with 0.1% crystal violet (Sigma-Aldrich, St. Louis, MO). Visible colonies were manually counted. Triplicate wells were measured for each treatment group.

**Cell cycle assays**

Flow cytometry assays were performed to analyze cell cycle progression. After ovarian cancer cells were incubated for 48 h, they were trypsinized and then fixed in 75% ethanol at −20 degrees Celsius overnight. Then cells were incubated with propidium iodide/RNase (BD Pharmingen, USA) for 15 min before the cell cycle distribution was analyzed.

**Invasion and cell wound-healing assays**

A cell invasion assay was performed using Transwell chamber inserts (8.0 mm, Corning, NY, USA) in a 24-well plate. A total of 2×10^4^ cells suspended in 200 µl of serum-free medium was added to each upper chamber; culture medium containing 20% FBS was placed in the bottom chambers. The cells were incubated for 24 or 48 h at 37°C. After incubation, the cells remaining on the upper surface were scraped off and washed away, whereas the cells on the lower surface were fixed with 20% methanol and stained with 0.1% crystal violet. The invaded cells in five randomly selected fields were counted under a microscope. In addition, we also conducted invasion assays by fluorescence staining. Briefly, cells in FBS-free medium (2×10^4^) were added to the top chamber with the bottom chamber containing 500 μL DMEM with 20% FBS. After 24 to 48 hours incubation, cells that invaded through the Matrigel and adhered to the lower surface of the filter were fixed with ethanol, stained with Hoechst 33342 (Life, USA), photographed at 40 ×, and counted in 10 different fields to determine the average number of cells at 200× under a microscope (BX51, Olympus, Japan). The experiments were repeated independently in triplicate.

For the wound-healing assay, cells were seeded in six-well plates and allowed to grow to 90-95% confluence. A single scratch wound was created at 6 h after transfection with siRNAs. The cells were washed with PBS to remove cell debris, supplemented with serum-free medium, and monitored. Images were acquired by phase-contrast microscopy at 0, 24 and 48 h after wounding. Besides, for eliminating the effect of cell proliferation, cells were pre-treated with medium containing 10 μg/mL of mitomycin c (S8146, Selleck Chemicals, Houston, TX, USA) for 2 h to block cell proliferation, and then subjected to cell wound-healing and invasion assays.

**RNA pulldown assays**

LINC00662 or antisense-LINC00662 RNAs were transcribed and labeled with Biotin RNA Labeling Mix (Roche, USA), treated with RNase-free DNase I (Takara, Japan) and purified using a RNeasy Mini Kit (QIAGEN, USA). Next, 1 pmol of biotinylated RNA was pretreated with RNA structure buffer (Beyotime Biotechnology, Shanghai, China) to obtain an appropriate secondary structure. The pretreated biotinylated RNAs were incubated with 1 mg of A2780 cellular protein extract at 4°C for 4 h, gently mixed with 40 μl of washed streptavidin beads (Thermo Fisher Scientific, Inc.) and incubated overnight on a rotator. Proteins binding to the biotin-labeled RNAs were precipitated and diluted in 60 μl of protein lysis buffer, separated by gel electrophoresis and visualized by silver staining. Specific bands (40-130 kDa) were excised for proteomic screening by mass spectrometry (Shanghai Applied Protein Technology, Shanghai, China). Protein annotations were retrieved from the human RefSeq protein database (National Center for Biotechnology Information (NCBI)) using Mascot version 2.4.01 (Matrix Science, London, UK). The retrieved proteins were confirmed by western blot analysis using two cell lines. The sequences of the primers used for *in vitro* transcription of LINC00662 and its truncated fragments are provided in Supplementary Table S6.

**RNA immunoprecipitation**

We performed RNA immunoprecipitation (RIP) experiments using a Magna RIP RNA-Binding Protein Immunoprecipitation Kit (Millipore, Billerica, MA, USA) according to the manufacturer's instructions and anti-HNRNPH1 and anti-GRP78 antibodies. Coprecipitated RNAs were detected by qRT-PCR. The sequences of the corresponding primers are provided in Supplementary Table S6.

**Immunoprecipitation**

An immunoprecipitation (IP) assay was performed using a Thermo Scientific™ Pierce Classic IP Kit (Thermo Fisher Scientific, MA, USA) according to the manufacturer’s instructions. A2780 and OVCAR-3 cells with LINC00662 knockdown or overexpression and negative control cells were treated with MG132. The cells were lysed in IP lysis/wash buffer containing protease inhibitors and an RNase inhibitor (Life Technologies) and were centrifuged at 14,000 × g for 15 min. The supernatants were incubated with Pierce Protein A/G Agarose overnight at 4°C. After washing, proteins were eluted with 2× nonreducing lane marker sample buffer containing 20 mM dithiothreitol (DTT) at 100°C for 5-10 min. The immunocomplexes were analyzed by SDS-PAGE and immunoblotting with anti-Flag, anti-AMFR, and anti-GRP78 antibodies.

**RNA sequencing**

RNA-seq was performed by Annoroad Co. (Hangzhou, China) using a HiSeq3000 system (Illumina, USA). For data processing, raw sequencing reads were aligned to the human reference genome (hg19) using the splice-aware aligner HISAT246. The read counts for each gene were normalized to fragments per kilobase of transcript per million mapped reads (FPKM) values. The cutoff used to determine differential gene expression was a |fold change (FC)| > 1.5 normalized to the corresponding negative control. The mean FPKM values of overlapping genes in the two subgroups of cells transfected with the two independent siRNAs were further analyzed. Kyoto Encyclopedia of Genes and Genomes (KEGG) and GSEA pathway analyses were performed.
**IC50 assays**

A2780 cells transfected with LINC00662 siRNAs were seeded in 96-well flat-bottom plates (2500 cells in 100 μl of cell suspensionper well) to determine the inhibitor concentrations that resulted in 50% inhibition of cell viability. We used a gradient of 6 concentrations according to the recommended concentrations of each inhibitor. After 96 h of culture, cell viability was evaluated using CCK-8 assays (Dojindo). Each experiment was performed with experimental triplicates and repeated three times.

**Immunohistochemical analysis**

Immunohistochemical analyses of OC specimens were conducted with specific antibodies as described previously [2]. Expression of proteins in the specimens was estimated according to the percentage and intensity of stained tumor cells. The staining percentage and intensity were graded as follows: 0 (0-4%), 1 (5-24%), 2 (25-49%), 3 (50-74%), or 4 (≥75%); and 0, 1, 2, or 3, respectively. The final score was obtained by multiplying the percentage and intensity scores. Tissue samples with final immunohistochemical scores of 0, 1-4, 5-8, and 9-12 were considered negative (-), weakly positive (+), moderately positive (++), and strongly positive (+++), respectively. All sections were individually scored by two investigators who were blinded to the clinical information of the OC patients.

***In vivo* assays**

Female athymic BALB/c nude mice, aged 4-6 weeks (16-20 g), were purchased from the Experimental Animal Center of Shanghai Cancer Institute (Shanghai, China). The mice (10 per group) were injected subcutaneously with 0.2 ml of cell suspension containing 8×10^6^ cells (stable negative control (NC)- and LINC00662-shRNA-mix-transfected A2780 cells) in the right axilla. Tumor growth rates were monitored. After a tumor became palpable, it was measured every other day, and its volume was calculated according to the following formula: volume = length × width^2^ × 0.5. The sample size was not predetermined for these experiments. For *In vivo* assays of LINC00662/GRP78 axis, 8×10^6^ cells sh-Control or LINC00662-shRNA-mix-A2780 cells in 200 μl of PBS were injected into the right axilla of 4-6 weeks old female BALB/c mice (Experimental Animal Center of Shanghai Cancer Institute, China). When the tumor volume reached 200 mm^3^, the mice (n=15) were randomly assigned into three groups (n = 5 mice/group) based on the treatment regimen: the sh-Control + PBS, LINC00662-shRNA-mix + PBS, LINC00662-shRNA-mix + HA15. HA15 (0.7 mg/mouse) or PBS was administered intraperitoneally on days 12, 16, 20, 24 and 28 post A2780 cell implantation. Tumor progression was monitored by assessment of the tumor volume and tumor weight.

We also used intraperitoneal injection model established as previously described to evaluate the peritoneal metastatic potential of human OC cell lines[3]. First, stable luciferase-labeled negative control- and LINC00662-shRNA-mix-transfected A2780 cells were generated, and 2×10^6^ cells in PBS were injected into the abdominal cavity of two groups of BALB/c mice. After 4 weeks, the development of peritoneal metastases was monitored by bioluminescence imaging (BLI, Xenogen IVIS 200 Imaging System), and then mice were sacrificed and the numbers of metastatic nodules were counted. All experiments were performed in accordance with relevant institutional and national guidelines and the regulations of the Shanghai Medical Experimental Animal Care Commission.

**Online tools**

The evaluation of prognostic values of lincRNAs were screened in Kaplan-Meier Plotter (http://kmplot.com/analysis/index.php?p=service) [4]. The coding potential of the LINC00662 transcript was analyzed using the Coding Potential Assessment Tool (CPAT, http://lilab.research.bcm.edu/cpat/index.php) and the Coding Potential Calculator (CPC, http://cpc.cbi.pku.edu.cn/). The correlation of expression between HNRNPH1 and LINC00662 were screened in GEPIA database (http://gepia.cancer-pku.cn/index.html) [5].

**References**

1 Li Z, Zhang J, Liu X, Li S, Wang Q, Di C *et al*. The LINC01138 drives malignancies via activating arginine methyltransferase 5 in hepatocellular carcinoma. *Nat Commun* 2018; 9: 1572.

2 Wang S, Li J, Xie J, Liu F, Duan Y, Wu Y *et al*. Programmed death ligand 1 promotes lymph node metastasis and glucose metabolism in cervical cancer by activating integrin beta4/SNAI1/SIRT3 signaling pathway. *Oncogene* 2018; 37: 4164-4180.

3 Shaw TJ, Senterman MK, Dawson K, Crane CA, Vanderhyden BC. Characterization of intraperitoneal, orthotopic, and metastatic xenograft models of human ovarian cancer. *Mol Ther* 2004; 10: 1032-1042.

4 Gyorffy B, Lanczky A, Szallasi Z. Implementing an online tool for genome-wide validation of survival-associated biomarkers in ovarian-cancer using microarray data from 1287 patients. *Endocr Relat Cancer* 2012; 19: 197-208.

5 Tang Z, Li C, Kang B, Gao G, Li C, Zhang Z. GEPIA: a web server for cancer and normal gene expression profiling and interactive analyses. *Nucleic Acids Res* 2017; 45: W98-W102.

**
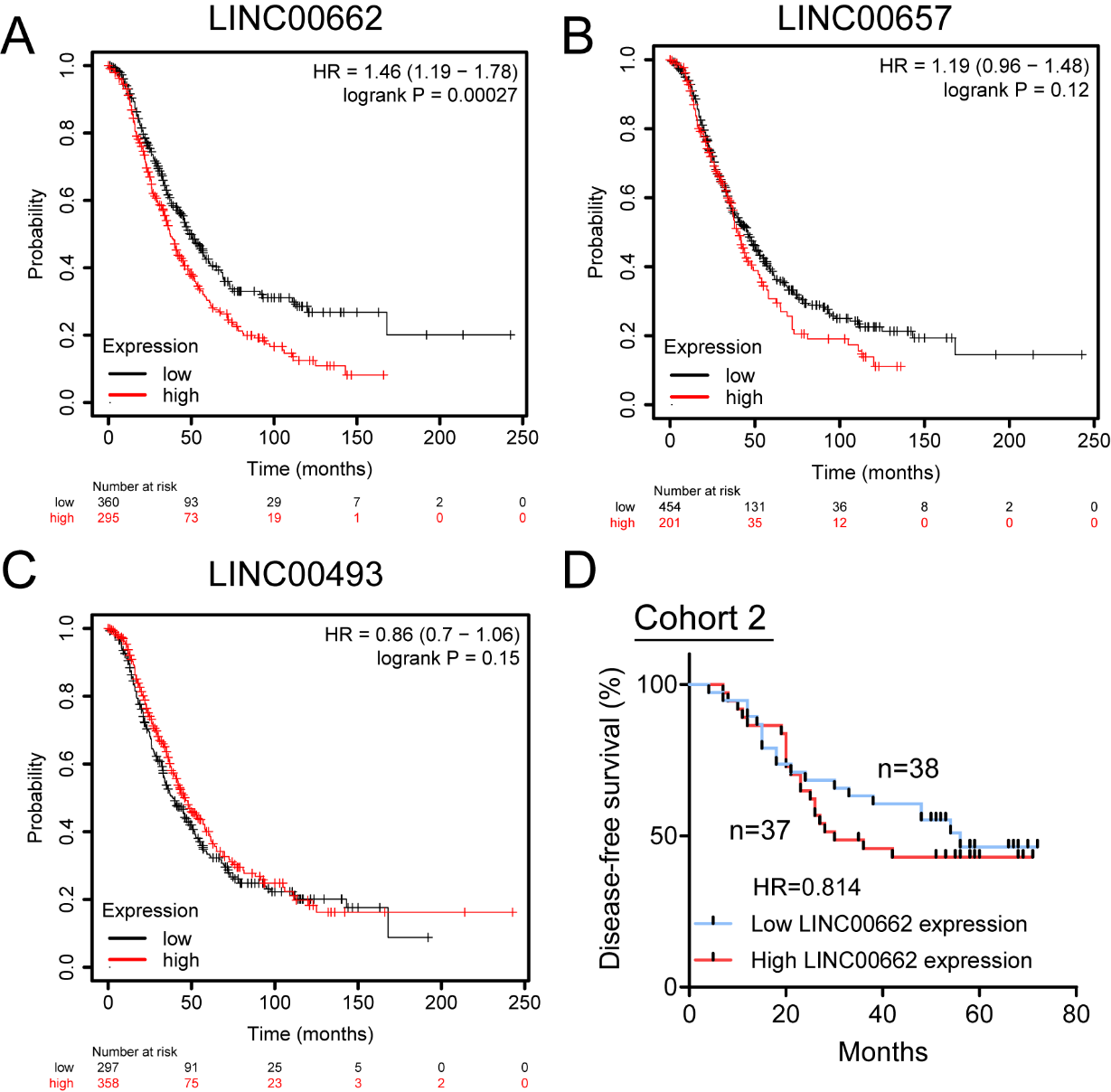
Supplementary Figures**

**Supplementary Figure S1. Screening of the prognostic value of lincRNAs in OC patients.**

(A-C) Kaplan-Meier analysis was performed to assess the associations between the expression of LINC00662, LINC00657, LINC00493 and the OS of patients with OC respectively in GEPIA database (P=0.00027, P=0.12, P=0.15). (D) LINC00662 expression did not correlate with DFS (P=0.511).

**
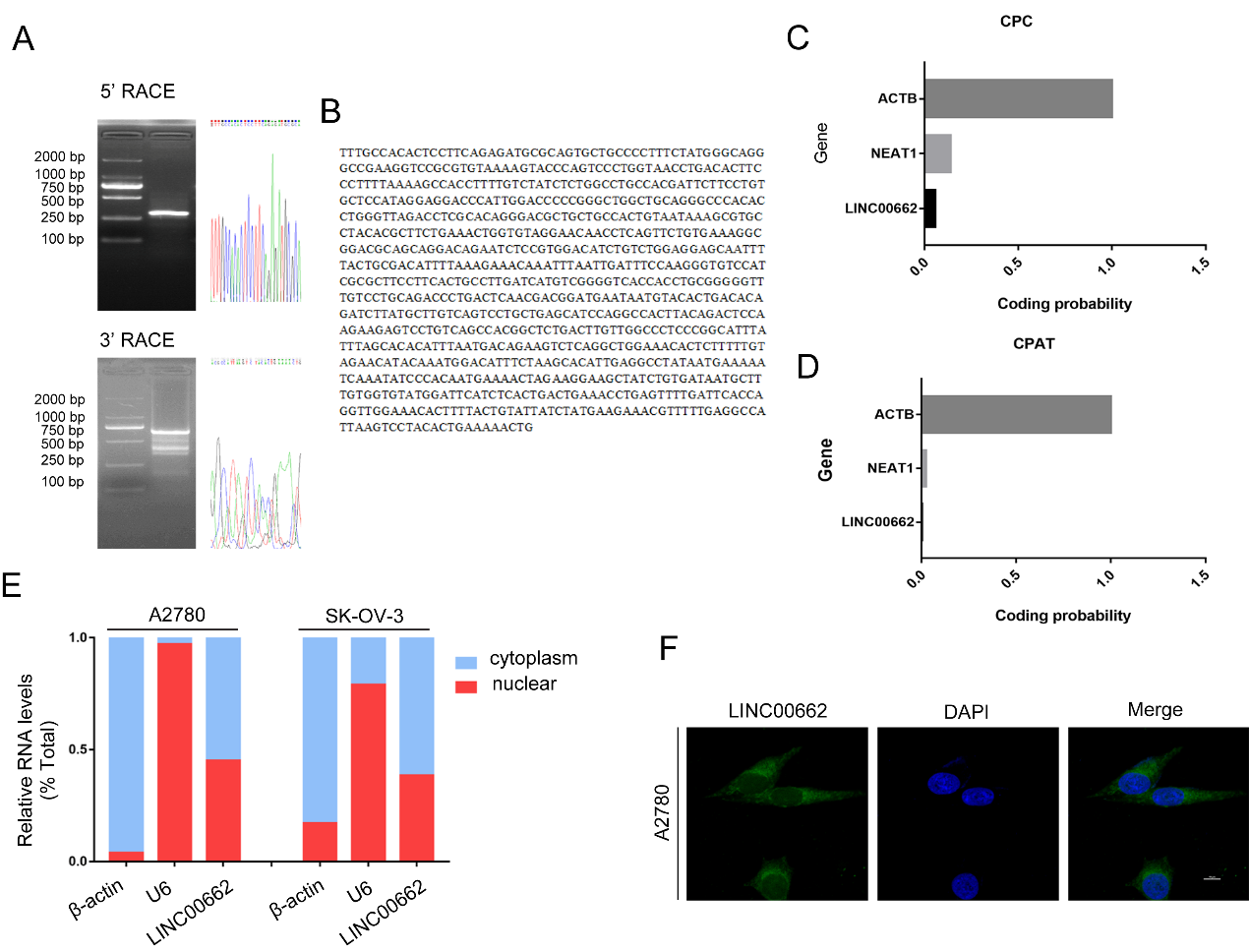
**

**Supplementary Figure S2. The characteristics of LINC00662 in OC.**

(A) Agarose gel electrophoresis of PCR products from the 5'-RACE and 3'-RACE procedure (left). Sequencing of RACE products is shown in the right panel. (B) The nucleotide sequence of full-length human LINC00662 in A2780 cells. (C-D) The protein coding potential of LINC00662 using the Coding Potential Calculator (CPC, http://cpc.cbi.pku.edu.cn/), Coding Potential Assessment Tool (CPAT, http://lilab.research.bcm.edu/cpat/). β-Actin served as the positive control of coding genes, and NEAT1 served as the negative control of noncoding genes. (E) The distributions of LINC00662 in A2780 and SK-OV-3 cells. β-Actin served as the cytoplasmic internal control, and U6 served as the nuclear internal control. (F) The location of LINC00662 using ISH in A2780 cells. Data are presented as means ± SEMs.

**
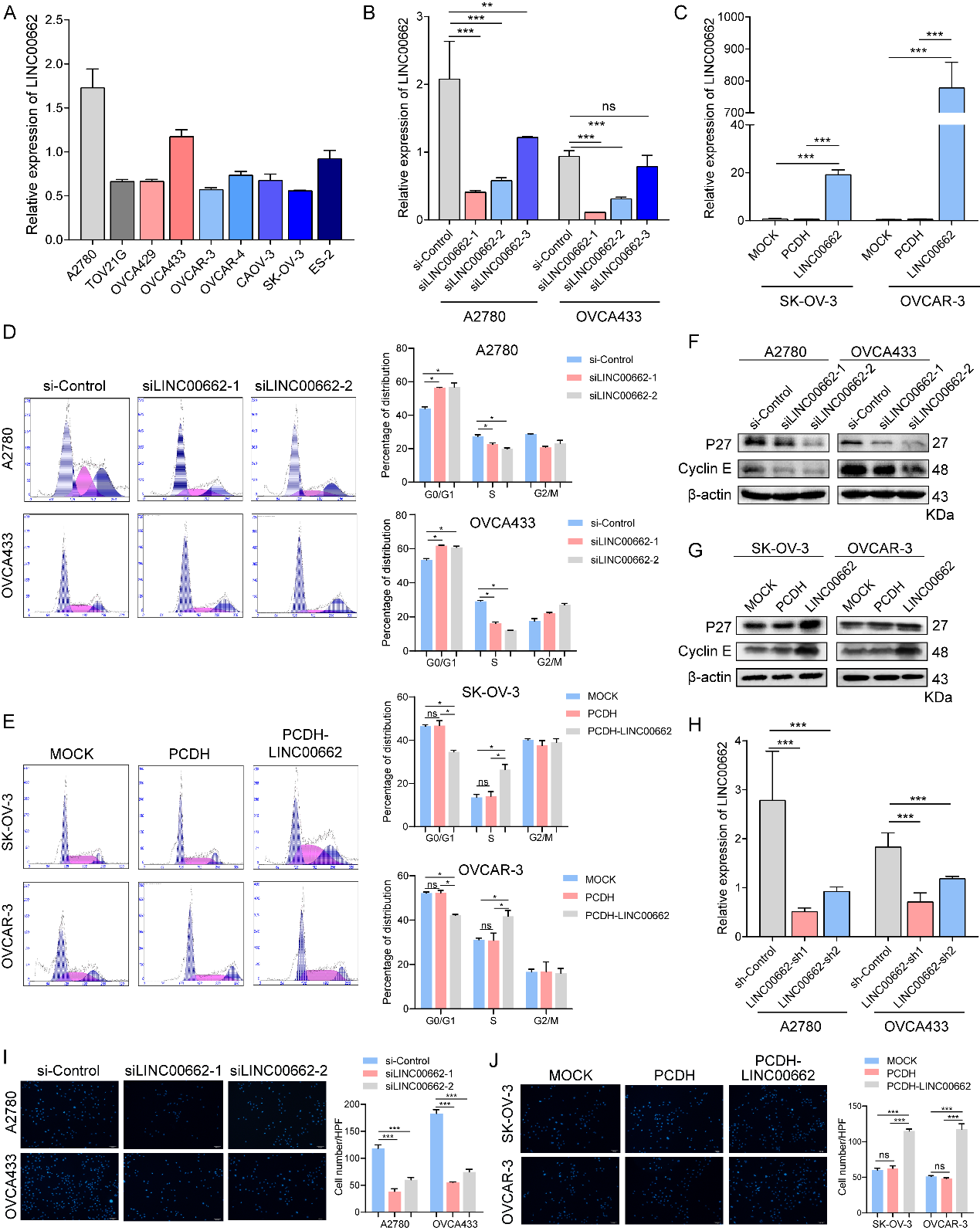
Supplementary Figure S3. LINC00662 promotes G1/S transition and invasion in OC cells.**

(A) Expression of LINC00662 in 9 OC cell lines. (B) qRT-PCR results of LINC00662 expression levels in A2780 and OVCA433 cells transiently transfected with siRNA. (C) qRT-PCR analysis of LINC00662 levels in SK-OV-3 and OVCAR-3 cells after stable LINC00662 overexpression. (D-E) Forty-eight hours after transfection, cell cycle was analyzed by flow cytometry in cells with LINC00662 knockdown or overexpression. (F-G) Immunoblot analysis of the expression levels of p27 and cyclin E in A2780 and OVCA433 cells transfected with LINC00662 siRNAs and in SK-OV-3 and OVCAR-3 cells overexpressing LINC00662. (H) qRT-PCR analysis of LINC00662 levels after stable LINC00662 downregulation in A2780 and OVCA433 cells prior to use in *in vivo* assays. (I-J) Representative images and the number of invaded cells per high-power field showed that cell invasiveness was suppressed by LINC00662 knockdown in A2780 and OVCA433 cells, but promoted by overexpression of LINC00662 in SK-OV-3 and OVCAR-3 cells. Values shown are means±SEMs. β-Actin was used as the internal control in F-G. A two-tailed Student’s t-test was used for statistical analysis. *P<0.05; **P<0.01; ***P<0.001. P<0.05 was considered significant. NS: not significant.

**
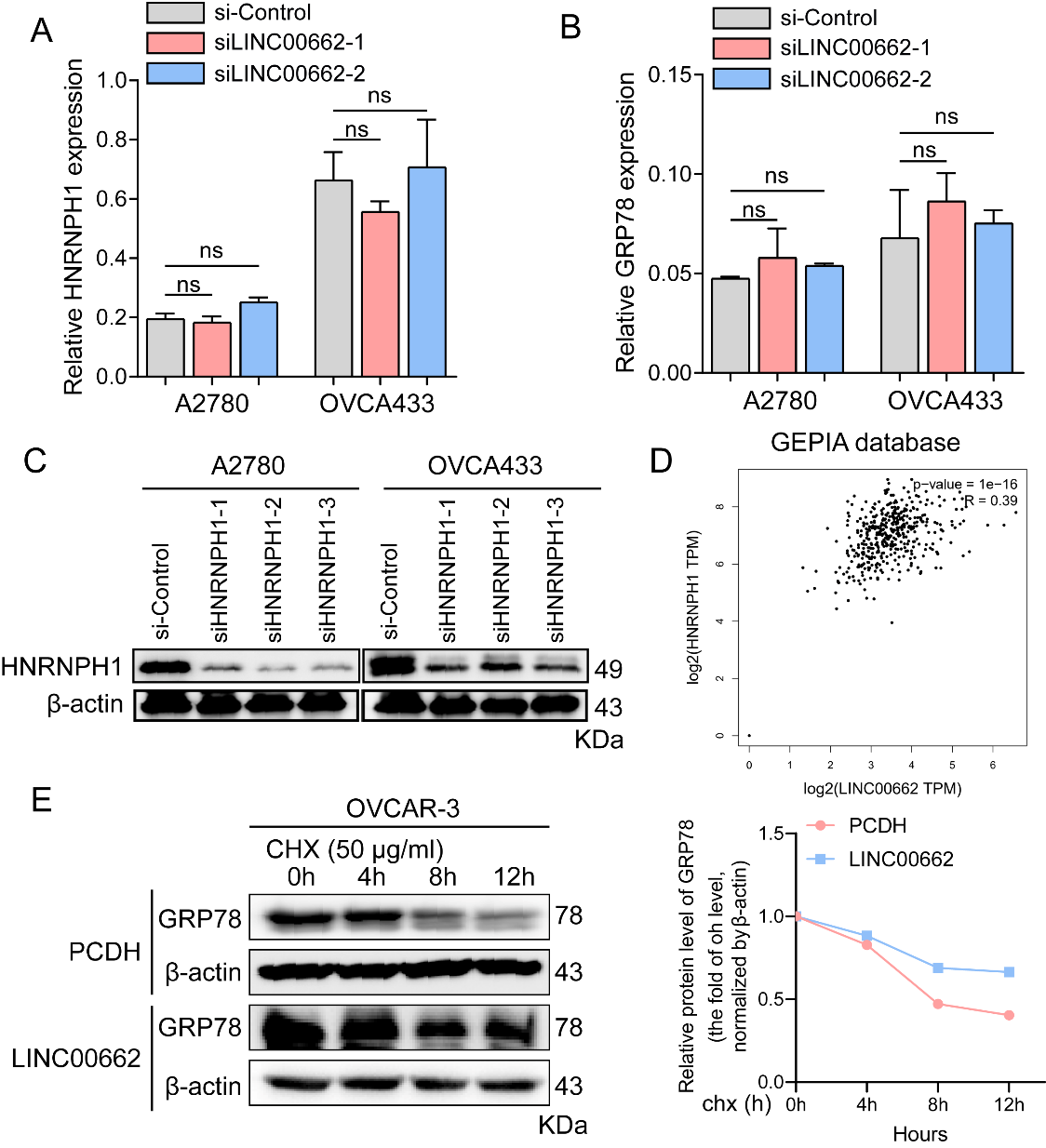
Supplementary Figure S4. The mechanism of LINC00662 interaction with HNRNPH1 and GRP78 in OC.**

(A-B) HNRNPH1 and GRP78 mRNA levels were quantified by qRT-PCR with LINC00662 knockdown in A2780 and OVCA433 cells. (C) Immunoblotting for protein levels of HNRNPH1 in A2780 and OVCA433 cells transfected with three independent HNRNPH1 siRNAs. (D) Correlation of the expression of HNRNPH1 and LINC00662 RNA levels in ovarian tumors from GEPIA database (r=0.39, *P*<0.001, http://gepia.cancer-pku.cn/index.html). (E) OVCAR-3 cells with LINC00662 overexpression, or the control cells were treated with cycloheximide (CHX, 50 μg/ml) for the indicated times. Left, immunoblotting for theGRP78 levels in whole-cell extracts; right, the densitometry analysis of the GRP78 protein levels; the relative fold of the level at 0 h normalized by β-actin. Values shown are means±SEMs. A two-tailed Student’s t-test was used for statistical analysis. P<0.05 was considered significant. NS: not significant.


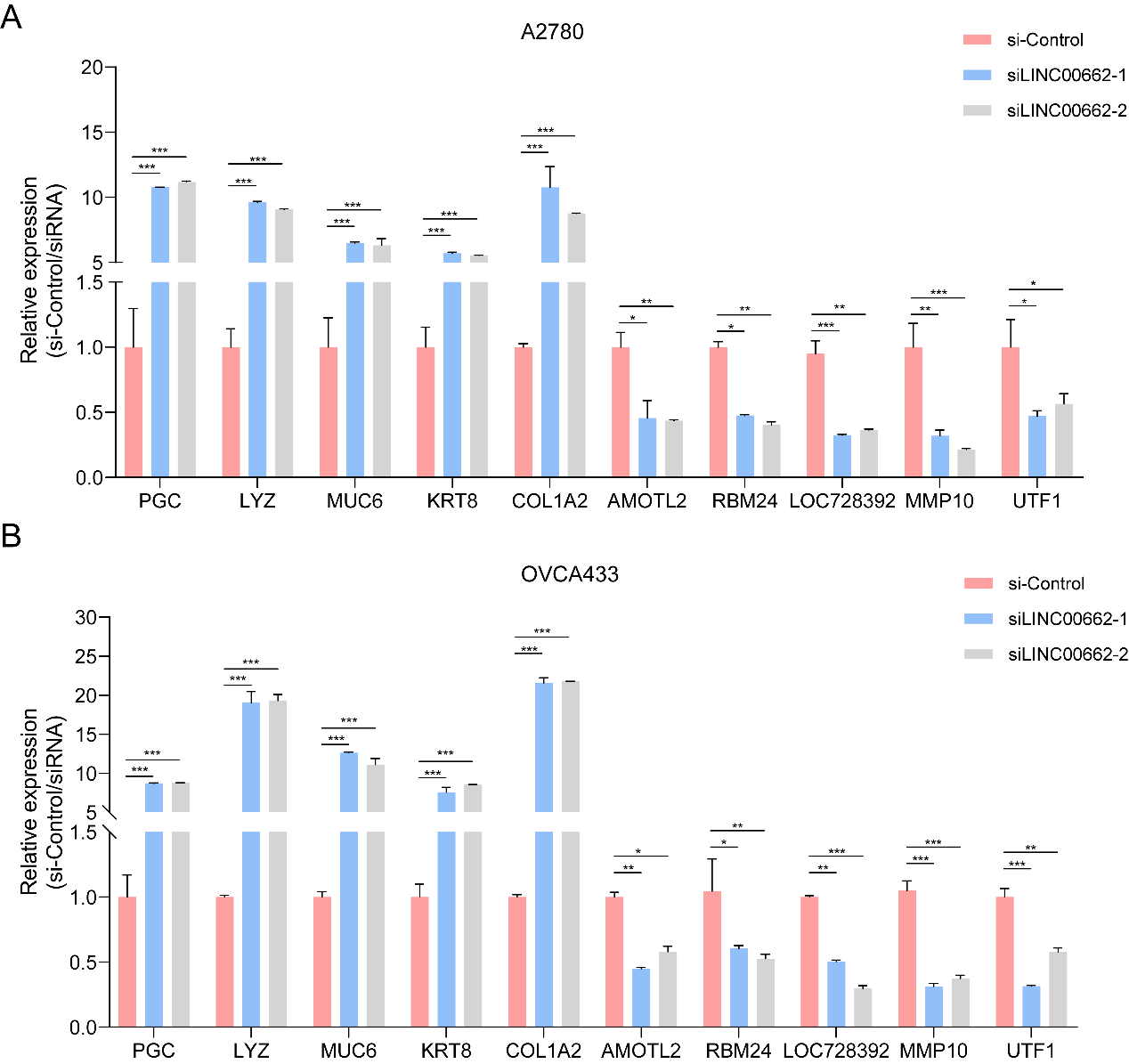
**Supplementary Figure S5. A few Top DEGs are** **randomly selected to validate the accuracy of RNA-seq dataset by RT-PCR.**

(A-B) The relative expression of PGC, LYZ, MUC6, KRT8, COL1A2, AMOTL2, RBM24, LOC728392, MMP10 and UTF1 in A2780 and OVCA433 cells transfected with si-Control or siRNAs targeted LINC00662. β-actin served as the internal control. Values were represented as the mean ± SEM in A-B. *P<0.05; **P<0.01; ***P<0.001. P<0.05 was considered significant.

**
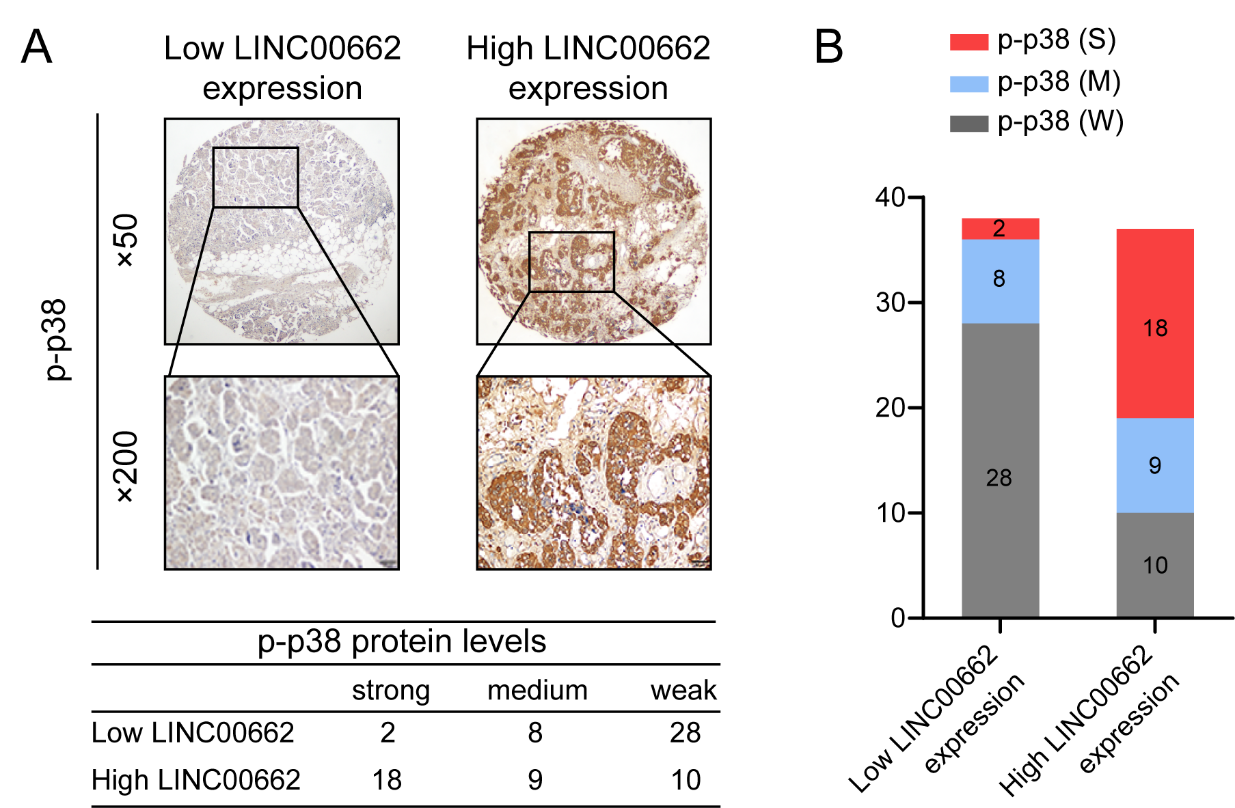
**

**Supplementary Figure S6.** **LINC00662 and p-p38 levels in clinical OC samples.**

(A) Representative immunohistochemical images of p-p38 in OC tissues (n=75) with low or high expression of LINC00662. (B) The chi-square test identified an association between LINC00662 and p-p38 levels in the OC samples (n = 75, p < 0.001).

**
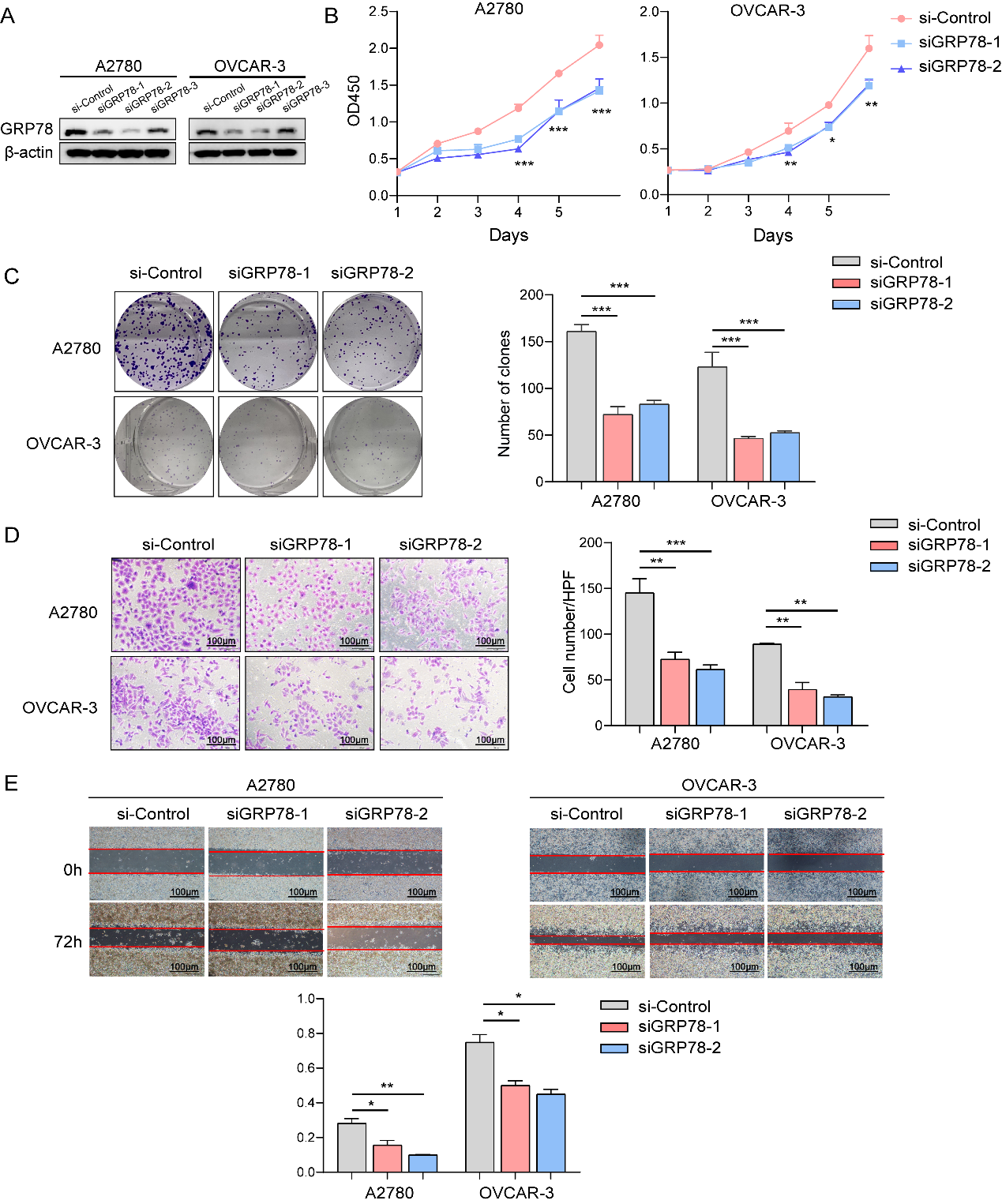
Supplementary Figure S7. GRP78 mediates OC cell growth and migration.**

(A) Immunoblotting for GRP78 in A2780 and OVCAR-3 cells transfected with three independent GRP78 siRNAs. (B) GRP78 knockdown impeded the effects of proliferation in A2780 and OVCAR-3 cells using a CCK-8 assay. (C) Representative images of colony formation ability in A2780 and OVCAR-3 cells treated with GRP78 siRNAs. (D) Representative images of Transwell experiments in A2780 and OVCAR-3 cells treated with GRP78 siRNA. 400× magnification. (E) Representative images of migration ability in A2780 and OVCAR-3 cells treated with GRP78 siRNA. Values shown are means±SEMs. A two-tailed Student’s t-test was used for statistical analysis. *P<0.05; **P<0.01; ***P<0.001. P<0.05 was considered significant.
