# Supplementary material for: HNRNPH1-stabilized LINC00662 promotes ovarian cancer progression by activating the GRP78/p38 pathway
Source: Oncogene. 2021 Jun 19;40(29):4770–82. doi: 10.1038/s41388-021-01884-5 (PMC8298204; doi:10.1038/s41388-021-01884-5)
Supplement: Supplementary file 2 — Supplementary Table S1 [file 41388_2021_1884_MOESM2_ESM.docx]

**Supplementary Table S1.** **Correlations between CNA and its RNA expression levels in TCGA ovarian cancer cohort.**

| Name | deletion count | application count | alteration frequency | correlation |
| --- | --- | --- | --- | --- |
| LINC00662 | 28 | 193 | 0.347747748 | 0.678264829 |
| LINC00657 | 17 | 218 | 0.392792793 | 0.560551416 |
| LINC00493 | 32 | 179 | 0.322522523 | 0.530736053 |
| LINC00240 | 54 | 178 | 0.320720721 | 0.454657498 |
| LINC00884 | 20 | 326 | 0.587387387 | 0.436205641 |
| LINC00888 | 6 | 354 | 0.637837838 | 0.405828827 |
| LINC00883 | 10 | 199 | 0.358558559 | 0.368938011 |
| LINC00654 | 29 | 218 | 0.392792793 | 0.366363495 |
| LINC00612 | 40 | 218 | 0.392792793 | 0.337675566 |
| LINC00652 | 31 | 177 | 0.318918919 | 0.333789357 |
| LINC00937 | 38 | 216 | 0.389189189 | 0.326813933 |
| LINC00942 | 39 | 241 | 0.434234234 | 0.295369079 |
| LINC00635 | 10 | 217 | 0.390990991 | 0.289896283 |
| LINC00886 | 6 | 319 | 0.574774775 | 0.286911295 |
| LINC00882 | 10 | 196 | 0.353153153 | 0.281797647 |
| LINC00636 | 10 | 217 | 0.390990991 | 0.26930435 |
| LINC00661 | 26 | 201 | 0.362162162 | 0.260692213 |
| LINC00336 | 53 | 170 | 0.306306306 | 0.257652314 |
| LINC00536 | 17 | 315 | 0.567567568 | 0.251213621 |
| LINC00904 | 52 | 182 | 0.327927928 | 0.247565524 |
| LINC00494 | 11 | 261 | 0.47027027 | 0.222425167 |
| LINC00303 | 20 | 195 | 0.351351351 | 0.203307998 |
| LINC00940 | 39 | 246 | 0.443243243 | 0.1906265 |
| LINC00658 | 29 | 218 | 0.392792793 | 0.18890681 |
| LINC00578 | 3 | 367 | 0.661261261 | 0.182011684 |
| LINC00885 | 22 | 325 | 0.585585586 | 0.181359677 |
| LINC00272 | 10 | 214 | 0.385585586 | 0.179092748 |
| LINC00184 | 20 | 231 | 0.416216216 | 0.165896828 |
| LINC00244 | 31 | 226 | 0.407207207 | 0.161505659 |
| LINC00880 | 4 | 320 | 0.576576577 | 0.155515585 |
| LINC00051 | 16 | 351 | 0.632432432 | 0.151688968 |
| LINC00941 | 15 | 211 | 0.38018018 | 0.136383526 |
| LINC00535 | 24 | 236 | 0.425225225 | 0.131199025 |
| LINC00518 | 57 | 212 | 0.381981982 | 0.128522498 |
| LINC00881 | 5 | 321 | 0.578378378 | 0.106436643 |
| LINC00477 | 26 | 221 | 0.398198198 | 0.102706926 |
| LINC00251 | 32 | 207 | 0.372972973 | 0.092078579 |
| LINC00534 | 35 | 217 | 0.390990991 | 0.089911939 |
| LINC00261 | 22 | 177 | 0.318918919 | 0.088132892 |
| LINC00851 | 34 | 181 | 0.326126126 | 0.086103795 |
| LINC00628 | 21 | 194 | 0.34954955 | 0.075715758 |
| LINC00659 | 11 | 303 | 0.545945946 | 0.073075516 |
| LINC00489 | 23 | 205 | 0.369369369 | 0.062173035 |
| LINC00588 | 18 | 202 | 0.363963964 | 0.056073877 |
| LINC00028 | 4 | 247 | 0.445045045 | 0.045722853 |
| LINC00887 | 21 | 330 | 0.594594595 | 0.031744086 |
| LINC00905 | 26 | 200 | 0.36036036 | 0.031047539 |
| LINC00626 | 6 | 213 | 0.383783784 | 0.023953828 |
| LINC00861 | 9 | 370 | 0.666666667 | 0.014044626 |
| LINC00906 | 22 | 206 | 0.371171171 | 0.013873823 |
| LINC00029 | 11 | 304 | 0.547747748 | 0.00475825 |
| LINC00488 | 12 | 208 | 0.374774775 | -0.028142785 |
| LINC00656 | 18 | 182 | 0.327927928 | -0.040811341 |
| LINC00582 | 19 | 204 | 0.367567568 | -0.044644362 |
| LINC00862 | 16 | 186 | 0.335135135 | -0.087301144 |
