# Supplementary material for: HNRNPH1-stabilized LINC00662 promotes ovarian cancer progression by activating the GRP78/p38 pathway
Source: Oncogene. 2021 Jun 19;40(29):4770–82. doi: 10.1038/s41388-021-01884-5 (PMC8298204; doi:10.1038/s41388-021-01884-5)
Supplement: Supplementary file 3 — Supplementary Table S2 [file 41388_2021_1884_MOESM3_ESM.docx]

**Supplementary Table S2. List of possible LINC00662-interacting proteins identified by Mass Spectrometry.**

| **Protein ID** | **Accession** | **-10lgP** | **Coverage (%)** | **Area Sample 2** | **#Peptides** | **#Unique** |
| --- | --- | --- | --- | --- | --- | --- |
| 19 | Q12906\|ILF3_HUMAN | 269.96 | 51 | 2.50E+07 | 36 | 30 |
| 21 | P19338\|NUCL_HUMAN | 252.14 | 58 | 2.16E+07 | 34 | 33 |
| 25 | Q92841\|DDX17_HUMAN | 239.94 | 36 | 5.98E+06 | 27 | 16 |
| 6 | Q9NR30\|DDX21_HUMAN | 239.87 | 35 | 7.99E+06 | 26 | 23 |
| 28 | Q02413\|DSG1_HUMAN | 231.65 | 26 | 9.95E+06 | 23 | 23 |
| 88 | P19013\|K2C4_HUMAN | 195.62 | 28 | 6.21E+05 | 22 | 6 |
| 55 | Q9Y2W1\|TR150_HUMAN | 228.26 | 26 | 7.73E+06 | 21 | 21 |
| 39 | P13646\|K1C13_HUMAN | 205.84 | 36 | 1.60E+06 | 21 | 6 |
| 47 | Q8N1N4\|K2C78_HUMAN | 195.03 | 28 | 1.76E+06 | 19 | 11 |
| 44 | P26599\|PTBP1_HUMAN | 218.58 | 44 | 1.30E+07 | 18 | 16 |
| 34 | Q5T749\|KPRP_HUMAN | 185.67 | 42 | 4.81E+06 | 18 | 18 |
| 4065 | Q92945\|FUBP2_HUMAN | 217.38 | 34 | 4.73E+06 | 16 | 16 |
| 119 | P36578\|RL4_HUMAN | 189.43 | 40 | 4.01E+06 | 15 | 15 |
| 45 | P11021\|GRP78_HUMAN | 200.28 | 27 | 2.29E+06 | 14 | 12 |
| 203 | Q9Y6M1\|IF2B2_HUMAN | 183.47 | 28 | 1.37E+06 | 13 | 10 |
| 72 | Q13435\|SF3B2_HUMAN | 171.47 | 20 | 9.16E+05 | 13 | 13 |
| 18 | P27816\|MAP4_HUMAN | 191.07 | 16 | 1.48E+06 | 12 | 12 |
| 81 | P61978\|HNRPK_HUMAN | 183.67 | 29 | 7.08E+06 | 12 | 11 |
| 120 | P11940\|PABP1_HUMAN | 181.87 | 22 | 1.14E+06 | 12 | 6 |
| 70 | P02768\|ALBU_HUMAN | 151.21 | 15 | 5.58E+06 | 12 | 12 |
| 486 | Q96AE4\|FUBP1_HUMAN | 168.67 | 20 | 1.16E+06 | 11 | 9 |
| 116 | P42285\|SK2L2_HUMAN | 158.95 | 12 | 7.68E+05 | 11 | 11 |
| 183 | P38159\|RBMX_HUMAN | 188.77 | 27 | 2.48E+06 | 10 | 10 |
| 395 | P51114\|FXR1_HUMAN | 162.72 | 22 | 1.15E+06 | 10 | 9 |
| 627 | Q9BUJ2\|HNRL1_HUMAN | 161.04 | 11 | 8.68E+05 | 9 | 8 |
| 4070 | Q08188\|TGM3_HUMAN | 155.16 | 17 | 4.55E+05 | 9 | 9 |
| 16 | P23246\|SFPQ_HUMAN | 150.44 | 16 | 5.80E+05 | 9 | 9 |
| 268 | P68363\|TBA1B_HUMAN | 165.52 | 24 | 1.90E+06 | 8 | 8 |
| 83 | O75533\|SF3B1_HUMAN | 146.38 | 11 | 2.64E+05 | 8 | 8 |
| 113 | Q9NZB2\|F120A_HUMAN | 142.18 | 12 | 7.77E+05 | 8 | 8 |
| 4073 | P31943\|HNRH1_HUMAN | 135.59 | 18 | 1.07E+06 | 8 | 6 |
| 4078 | Q15637\|SF01_HUMAN | 121.9 | 11 | 1.19E+06 | 8 | 8 |
