# Supplementary material for: HNRNPH1-stabilized LINC00662 promotes ovarian cancer progression by activating the GRP78/p38 pathway
Source: Oncogene. 2021 Jun 19;40(29):4770–82. doi: 10.1038/s41388-021-01884-5 (PMC8298204; doi:10.1038/s41388-021-01884-5)
Supplement: Supplementary file 4 — Supplementary Table S3 [file 41388_2021_1884_MOESM4_ESM.docx]

**Supplementary Table S3. RNA-seq analyses of the differentially gene expression profile affected by LINC00662 knockdown**

| GENE | logFC | AveExpr |
| --- | --- | --- |
| PGC | 6.51543002 | 5.171355655 |
| LYZ | 3.24925893 | 3.270714041 |
| MUC6 | 2.951857909 | 2.770356521 |
| KRT8 | 2.750847122 | 3.774503601 |
| COL1A2 | 2.629642944 | 2.685940682 |
| CD74 | 2.53770665 | 3.349438007 |
| HIST1H4H | 2.469512235 | 2.757401748 |
| CA2 | 2.464857103 | 2.564997087 |
| CAPN8 | 2.363207597 | 2.327641976 |
| MUC13 | 2.355938026 | 2.264785262 |
| PSCA | 2.238665838 | 3.352241064 |
| VAMP7 | 2.176177808 | 2.552858106 |
| ANXA10 | 2.051633316 | 2.174163652 |
| CLDN18 | 2.011839027 | 3.211128678 |
| TOMM22 | 1.962823081 | 6.737547604 |
| S100A6 | 1.866581716 | 4.888570086 |
| CXCL17 | 1.836045196 | 1.955470861 |
| KRT18 | 1.803042284 | 3.12169346 |
| HIST1H4E | 1.72994066 | 2.261683509 |
| A2M | 1.725309139 | 1.960482311 |
| LGALS4 | 1.724874197 | 3.117642317 |
| TPM4 | 1.692209603 | 8.056015882 |
| HIST1H2BD | 1.689445466 | 4.607099371 |
| HABP4 | 1.686828855 | 4.406081024 |
| AKR1C3 | 1.653571681 | 2.727319239 |
| TSPAN1 | 1.645655504 | 3.463153907 |
| CLPSL2 | 1.550044713 | 2.646998319 |
| NOLC1 | 1.535758713 | 6.648514278 |
| GANAB | 1.522537724 | 8.582023814 |
| C7orf55 | 1.514315961 | 3.338181141 |
| EMC6 | 1.467571636 | 5.636087319 |
| MED11 | 1.438129949 | 4.831359795 |
| RGS19 | 1.415770871 | 3.753336949 |
| B4GALT1 | 1.413782417 | 5.462588822 |
| RAB25 | 1.409301424 | 1.592876414 |
| COLEC11 | 1.393776634 | 4.198135281 |
| OGFR | 1.373621089 | 4.939771202 |
| SDC4 | 1.360019798 | 4.930092891 |
| CCDC80 | 1.315477219 | 5.171670364 |
| BICD2 | 1.310777978 | 4.200455109 |
| HDGFRP2 | 1.306496067 | 7.068304438 |
| MEOX1 | 1.304615762 | 5.232376874 |
| NPPA | 1.303277249 | 1.599230488 |
| LOC100652807 | 1.283674763 | 1.957434874 |
| SETD7 | 1.259780301 | 4.284333623 |
| CYBA | 1.25436494 | 2.222991308 |
| NES | 1.249025825 | 7.77632596 |
| FAM32A | 1.248073654 | 6.25753051 |
| MFSD3 | 1.24348805 | 4.823167228 |
| SLC33A1 | 1.235605869 | 5.140515501 |
| LOC101928852 | 1.226368454 | 1.632189971 |
| GOLGA2 | 1.212993338 | 6.099428767 |
| HIST1H4D | 1.210289982 | 1.931841319 |
| ADPRHL1 | 1.196652988 | 5.212252884 |
| C19orf68 | 1.196561908 | 3.815599251 |
| RAD23A | 1.193306171 | 6.98880745 |
| FAM210B | 1.191063537 | 4.626832992 |
| POLR3GL | 1.183446997 | 5.554696353 |
| SLC44A4 | 1.181991086 | 1.81530513 |
| HIST1H3E | 1.175197137 | 4.336416894 |
| FAAH | 1.170577568 | 1.445587821 |
| R3HCC1 | 1.170197727 | 4.461512681 |
| SPINT1 | 1.166526869 | 1.523883706 |
| IL18 | 1.166465054 | 2.84903913 |
| PFN4 | 1.16625112 | 3.006499867 |
| CAPN6 | 1.163494559 | 2.422406765 |
| SLC16A8 | 1.161437705 | 2.088839342 |
| PACRG | 1.160536044 | 1.666707164 |
| ZNF616 | 1.157839388 | 3.475189444 |
| COL1A1 | 1.154175256 | 4.422249294 |
| FAM228B | 1.149213236 | 4.539910032 |
| MMP14 | 1.146369804 | 2.112582156 |
| FAM133A | 1.135991394 | 3.857314267 |
| AKR7A3 | 1.130637299 | 2.12193533 |
| C9orf78 | 1.129978328 | 4.976583113 |
| HLA-DPA1 | 1.129866256 | 1.476977289 |
| GNG8 | 1.126973324 | 3.21486616 |
| CASP7 | 1.126423442 | 4.816766862 |
| LIPH | 1.125586506 | 1.808797503 |
| CDK14 | 1.114270309 | 2.677238462 |
| HIST3H2A | 1.111727454 | 5.640477331 |
| ARHGAP30 | 1.107924551 | 2.798080811 |
| HIST1H3F | 1.105306273 | 1.735277415 |
| SGCA | 1.103155757 | 5.648244814 |
| DQX1 | 1.101689471 | 3.194568684 |
| WFDC3 | 1.096796627 | 1.411253916 |
| ZNF629 | 1.089459604 | 5.164605526 |
| KLK1 | 1.086522937 | 1.83857412 |
| ACTA2 | 1.084737073 | 3.605357029 |
| TMEM190 | 1.081810158 | 1.975623673 |
| CAPG | 1.079217568 | 2.957852855 |
| C4orf33 | 1.074056864 | 4.532339429 |
| ZCCHC3 | 1.073974315 | 6.34430013 |
| PRDX4 | 1.062357181 | 6.694540032 |
| CHST14 | 1.055574493 | 5.726517481 |
| DUSP19 | 1.050606269 | 1.948859651 |
| LOC100129083 | 1.050374401 | 1.501240333 |
| FN1 | 1.04924183 | 1.77163799 |
| PTGS2 | 1.047931487 | 2.137949022 |
| AMPD3 | 1.047312381 | 2.340462343 |
| VILL | 1.04721426 | 1.900131159 |
| BMS1 | 1.043608424 | 5.300113607 |
| FOXP4 | 1.040402781 | 4.329574656 |
| SYCE3 | 1.038590315 | 1.71559313 |
| PPP1R14B | 1.030624004 | 6.959928997 |
| CLDN7 | 1.029009042 | 2.406021755 |
| C11orf94 | 1.027895834 | 1.804802183 |
| LAD1 | 1.023013469 | 1.592426737 |
| SLC7A8 | 1.020144457 | 1.414900728 |
| DENND5A | 1.018910646 | 6.435784931 |
| TMEM176A | 1.015341028 | 1.628389141 |
| LOC102724296 | 1.008514897 | 3.355791493 |
| RERGL | 1.002880904 | 3.886092109 |
| TOP1MT | 1.000520375 | 5.444153793 |
| ENSA | 0.996724661 | 6.932336222 |
| FKBP5 | 0.996214948 | 5.579408691 |
| GTF2F2 | 0.996136397 | 5.512171061 |
| ZNF664 | 0.995797791 | 6.277997864 |
| COQ7 | 0.995774998 | 3.715031543 |
| PDCL3 | 0.995406802 | 5.531289287 |
| PLA2G6 | 0.987233595 | 5.174892274 |
| DUOX2 | 0.986720246 | 1.988995462 |
| PRR4 | 0.985483865 | 2.224015445 |
| SEC13 | 0.984945865 | 6.470698897 |
| PALM3 | 0.983811093 | 3.635318522 |
| ZW10 | 0.981460537 | 4.56217563 |
| ZNF267 | 0.980757833 | 4.46220117 |
| IFT27 | 0.976000059 | 5.502517374 |
| XRCC4 | 0.973861288 | 3.985146354 |
| WBSCR17 | 0.973086061 | 3.930985959 |
| LOC646730 | 0.968889474 | 2.109595883 |
| DPCR1 | 0.960567098 | 1.430224787 |
| MRFAP1 | 0.959757164 | 8.090900975 |
| ANXA4 | 0.955428576 | 4.46112822 |
| LOC102723902 | 0.94958534 | 4.376143269 |
| APOBEC2 | 0.945224272 | 1.939732813 |
| NDRG1 | 0.944660028 | 2.260751059 |
| HIST1H3D | 0.942925503 | 2.718798055 |
| ARHGEF9 | 0.938298376 | 4.19674943 |
| SLC5A5 | 0.935518681 | 3.53937786 |
| LOC730268 | 0.931168752 | 2.52277977 |
| MAD1L1 | 0.928346135 | 4.594928917 |
| ZNF165 | 0.927277177 | 2.145954384 |
| ZNF671 | 0.92709429 | 1.370325729 |
| LOC388210 | 0.926679986 | 1.768472594 |
| PIR | 0.926494544 | 4.892207108 |
| C14orf178 | 0.92460123 | 1.470365594 |
| CREB3L4 | 0.922129487 | 4.392178787 |
| POLR2D | 0.920593464 | 5.522225882 |
| PLA2G4A | 0.91989272 | 4.725537806 |
| BCL7B | 0.919758883 | 5.704699051 |
| MAPK6 | 0.91641623 | 5.763143058 |
| SERPINB6 | 0.915105986 | 5.685110755 |
| ATP8B1 | 0.914957318 | 1.901254203 |
| SLC27A6 | 0.912959294 | 1.599790629 |
| ELF3 | 0.912292283 | 1.807769133 |
| PLA2G12A | 0.911569399 | 4.353356722 |
| GPT | 0.909077698 | 3.220509567 |
| AC022007.5 | 0.908801909 | 2.435557432 |
| CREB3L2 | 0.906478766 | 4.878272206 |
| FOPNL | 0.904169504 | 6.127817909 |
| LOC100996693 | 0.901438096 | 2.806557225 |
| IL21R | 0.8995082 | 2.411820586 |
| CAMLG | 0.899153148 | 5.617417406 |
| SRGN | 0.896083369 | 1.464369237 |
| AES | 0.895480552 | 7.929914698 |
| COL6A3 | 0.892742891 | 2.279488897 |
| CPS1 | 0.891659911 | 3.92777529 |
| THOC7 | 0.88877996 | 6.503879726 |
| WBP4 | 0.887605705 | 4.736191712 |
| LACE1 | 0.887370655 | 2.85460933 |
| ZKSCAN1 | 0.884896706 | 5.297639683 |
| GALNT11 | 0.881179096 | 5.347963353 |
| NUDCD3 | 0.878824119 | 4.933837355 |
| PSKH1 | 0.878237958 | 5.214112912 |
| HSDL2 | 0.877335253 | 5.991328227 |
| HIST1H2BF | 0.876988275 | 2.368858248 |
| TTC33 | 0.875913393 | 3.785362237 |
| PERP | 0.87555821 | 4.560524367 |
| CSRP1 | 0.874544182 | 3.615660574 |
| MORN4 | 0.872492733 | 4.3520956 |
| STX12 | 0.871699855 | 4.804488217 |
| OSGEP | 0.870744384 | 4.841972652 |
| LOC102724164 | 0.870692903 | 3.857974916 |
| QTRT1 | 0.866570007 | 5.398024388 |
| CAPN9 | 0.866349441 | 1.321859488 |
| GPX3 | 0.866315586 | 1.71989722 |
| ZFAND1 | 0.865473109 | 5.657441902 |
| MFAP4 | 0.865040438 | 1.953078699 |
| HYAL1 | 0.86503966 | 3.912598602 |
| C11orf71 | 0.864490404 | 4.193031614 |
| WDR54 | 0.861776259 | 6.874674254 |
| EFCAB9 | 0.860454335 | 1.717000262 |
| FMO4 | 0.859544153 | 3.317981305 |
| EHHADH | 0.859444804 | 1.417612838 |
| C9orf50 | 0.857205015 | 1.887819671 |
| C17orf50 | 0.856545757 | 1.729906357 |
| NR1H3 | 0.855712709 | 4.226150381 |
| TRAPPC9 | 0.855230752 | 4.069906969 |
| CALR | 0.85417026 | 10.72658226 |
| ATG4A | 0.851856303 | 4.190901057 |
| ATP6V1G2 | 0.851225637 | 3.330036297 |
| MPHOSPH10 | 0.85026681 | 5.257168558 |
| PNMAL2 | 0.849437798 | 1.933998495 |
| ANAPC15 | 0.848989826 | 5.232591076 |
| PHF5A | 0.848962998 | 6.089261798 |
| NASP | 0.848716525 | 7.80493135 |
| FAM46C | 0.846542206 | 1.260636488 |
| HYPK | 0.844815097 | 4.53817467 |
| PPP1R3C | 0.843715886 | 5.229486185 |
| TMEM27 | 0.842839228 | 1.50719414 |
| FGFBP3 | 0.842149561 | 3.650269931 |
| ZNF565 | 0.841440483 | 2.335863299 |
| TNNC2 | 0.841002265 | 1.391317974 |
| TMEM39A | 0.840740013 | 4.596042206 |
| ZNF618 | 0.840623431 | 4.15407825 |
| PTK2B | 0.840326301 | 3.42006083 |
| MAPK10 | 0.839952788 | 1.980114418 |
| NPEPL1 | 0.838497541 | 2.583109202 |
| C19orf82 | 0.837717011 | 3.166768176 |
| KIF1C | 0.837421442 | 5.282982365 |
| CLIC3 | 0.837150278 | 3.631110289 |
| MISP | 0.837007093 | 1.657907594 |
| TFAP4 | 0.835119944 | 5.091781974 |
| UBQLNL | 0.834760595 | 1.328546214 |
| SLC24A1 | 0.834079536 | 4.068801164 |
| FAIM | 0.83311794 | 3.701021096 |
| CAPN3 | 0.832533132 | 2.567710681 |
| PLEKHA6 | 0.831834563 | 1.461917395 |
| WBSCR16 | 0.831035856 | 6.478404317 |
| ZDHHC16 | 0.831009665 | 5.680416756 |
| DPM3 | 0.830664561 | 6.859353536 |
| LOC102724285 | 0.829841141 | 2.87838859 |
| SERPINI1 | 0.827972555 | 3.91854449 |
| COL3A1 | 0.827191936 | 9.276115637 |
| SLC25A6 | 0.82604437 | 5.489448227 |
| TNFSF13 | 0.825781548 | 2.340094141 |
| DHX37 | 0.825620249 | 4.343473606 |
| MUC3A | 0.824153635 | 1.569760325 |
| MARVELD1 | 0.823696585 | 5.478410754 |
| HS2ST1 | 0.82285877 | 5.405363595 |
| FAM159B | 0.822367063 | 2.5863183 |
| TBC1D10B | 0.821760348 | 5.376742106 |
| MGMT | 0.818270188 | 4.818437281 |
| TM4SF19 | 0.818200676 | 2.165230849 |
| C5orf55 | 0.817734188 | 2.743770601 |
| UNC45A | 0.815678248 | 5.700329426 |
| C1RL | 0.81532391 | 5.421153945 |
| HIST1H2AD | 0.813799752 | 1.50100805 |
| TFPI | 0.81322761 | 2.309402033 |
| ECI1 | 0.810987082 | 7.609219981 |
| SPRYD4 | 0.809030289 | 4.273775945 |
| ADAM15 | 0.807032172 | 6.718102827 |
| MZB1 | 0.806741857 | 1.282607117 |
| COL12A1 | 0.806596303 | 1.31288618 |
| CYP3A5 | 0.806251353 | 1.985273918 |
| ME1 | 0.803246222 | 3.970002622 |
| MESDC2 | 0.801530633 | 4.720860882 |
| GPAA1 | 0.800839835 | 6.233382055 |
| GTF2H3 | 0.799854528 | 5.514955876 |
| CSRP2BP | 0.799213149 | 4.146056546 |
| C1orf56 | 0.797480819 | 4.142953093 |
| PRMT6 | 0.796364898 | 5.000846928 |
| CT83 | 0.796017048 | 4.019447887 |
| HIRIP3 | 0.796004029 | 4.017978354 |
| DCDC2B | 0.795912083 | 1.701723323 |
| DZIP3 | 0.792244753 | 5.409850607 |
| PPM1M | 0.790819504 | 4.072077329 |
| KCTD19 | 0.788848753 | 2.718862269 |
| ALDH3A1 | 0.788654647 | 1.289729188 |
| HIST1H2AK | 0.788162616 | 3.335463713 |
| MYO1D | 0.786917549 | 2.502810525 |
| PDXP | 0.786634825 | 6.566089429 |
| COQ10A | 0.784926615 | 5.689279166 |
| PET117 | 0.783077132 | 3.022603944 |
| PCK2 | 0.781877101 | 6.578373529 |
| FAM78A | 0.780833004 | 4.218917663 |
| CCDC9 | 0.77871355 | 4.616228227 |
| ZNF248 | 0.778435668 | 4.571404997 |
| LGALS3 | 0.777979362 | 2.130182179 |
| MGLL | 0.777719973 | 1.227173161 |
| MAMSTR | 0.777647239 | 5.016553458 |
| HPGD | 0.777033794 | 1.305101062 |
| MYH11 | 0.775905997 | 2.291845319 |
| EPCAM | 0.775864165 | 3.162966617 |
| ANXA6 | 0.774964394 | 7.044538643 |
| EPHA3 | 0.774800805 | 3.11181231 |
| SLC9A9 | 0.774503793 | 2.782327443 |
| CA7 | 0.772339123 | 2.729086269 |
| ACO2 | 0.771381647 | 6.602536313 |
| PPIP5K2 | 0.771311343 | 5.04721326 |
| TBC1D9 | 0.770774128 | 4.197379855 |
| CYCS | 0.769220729 | 5.57535146 |
| BIRC7 | 0.768146907 | 1.817851916 |
| RPL26L1 | 0.767402724 | 5.64606163 |
| HDAC1 | 0.765302627 | 8.055719832 |
| TMEM30A | 0.764381904 | 5.993085733 |
| LYRM9 | 0.764311767 | 3.753137176 |
| RNF43 | 0.763805292 | 2.163880028 |
| GBP3 | 0.763263591 | 1.168429687 |
| PACSIN1 | 0.762764077 | 3.096502162 |
| TAS2R3 | 0.762081179 | 1.810701095 |
| LCN15 | 0.761940098 | 1.715533732 |
| LRRC4B | 0.761523022 | 4.157846562 |
| LONP1 | 0.761341068 | 7.991499847 |
| ITGA1 | 0.760606087 | 1.717415756 |
| C8G | 0.759966684 | 1.724423662 |
| SNAPC1 | 0.75970866 | 5.317077589 |
| PDLIM1 | 0.759371253 | 4.081941876 |
| C9orf129 | 0.757831133 | 1.406028983 |
| DERL3 | 0.756242408 | 2.161051418 |
| HIST1H2AM | 0.755238024 | 2.825680267 |
| PPP1R3E | 0.754069808 | 4.91228259 |
| SFXN2 | 0.754002085 | 4.504012142 |
| VOPP1 | 0.753603154 | 4.861965379 |
| NUDT13 | 0.753526079 | 3.644455183 |
| FBXO16 | 0.752597828 | 4.357997851 |
| PAFAH1B2 | 0.751263765 | 6.458911888 |
| SLC39A7 | 0.750934782 | 7.330805944 |
| FAM218A | 0.75078213 | 1.632797218 |
| CBLN3 | 0.749022706 | 3.515425698 |
| ZBTB17 | 0.749019593 | 4.83088443 |
| ODF3B | 0.748994047 | 3.106841583 |
| HSD17B8 | 0.748544839 | 5.039560228 |
| C1orf162 | 0.748035768 | 1.906860845 |
| TAF3 | 0.747927528 | 3.395062374 |
| BECN1 | 0.746914952 | 5.827913451 |
| DENR | 0.746590156 | 5.533571992 |
| CDRT4 | 0.744674898 | 3.290336389 |
| ANXA7 | 0.743086339 | 6.341858084 |
| PLEKHA2 | 0.741989995 | 5.330158535 |
| KCNRG | 0.74077943 | 3.040317847 |
| GLB1L | 0.738238957 | 3.03356833 |
| BLMH | 0.736827924 | 5.529809263 |
| ICA1 | 0.735856198 | 3.840209295 |
| ALDH1A1 | 0.735184247 | 1.479792193 |
| HIST1H1B | 0.734749144 | 1.572203751 |
| G3BP2 | 0.734201379 | 6.209582494 |
| TAF15 | 0.73356805 | 7.320681521 |
| SLC46A3 | 0.733519994 | 3.292013877 |
| GNPDA1 | 0.732468944 | 4.637307929 |
| RBM47 | 0.731732041 | 1.568951599 |
| PFKL | 0.729682901 | 7.352734769 |
| PBDC1 | 0.729595595 | 5.397461503 |
| IGSF23 | 0.729558776 | 1.333304238 |
| MRM1 | 0.729118316 | 3.700559297 |
| MIPEP | 0.729103423 | 4.821857051 |
| PATL2 | 0.728983671 | 2.025730911 |
| CYB5RL | 0.728763218 | 3.318029093 |
| PTPN11 | 0.727837121 | 6.591870534 |
| CTF1 | 0.72651988 | 5.617043849 |
| SRP19 | 0.725888162 | 5.309117317 |
| UBE2Q2L | 0.725635818 | 2.385757814 |
| KDM2B | 0.725480155 | 5.445425794 |
| EIF3F | 0.725371019 | 8.562775035 |
| NDUFS2 | 0.724546635 | 7.633693257 |
| CYB5R2 | 0.723741713 | 1.663180101 |
| ZNF467 | 0.723738537 | 6.378321729 |
| AEBP1 | 0.723633267 | 1.156020672 |
| HSPB7 | 0.723533841 | 3.755162681 |
| TVP23C-CDRT4 | 0.722558399 | 3.442346304 |
| SCFD2 | 0.721306875 | 2.684591346 |
| GZMM | 0.719117362 | 2.837314524 |
| SH2D3A | 0.717444998 | 1.86876272 |
| SUGCT | 0.716096891 | 3.453451649 |
| ERLIN2 | 0.716045436 | 4.701147864 |
| SF3B2 | 0.715944447 | 7.811261015 |
| LOC101929097 | 0.714910898 | 2.330324577 |
| CENPBD1 | 0.714864547 | 3.349021031 |
| ZNF18 | 0.713519176 | 3.218201637 |
| GTF3A | 0.712485555 | 6.995641911 |
| S100A2 | 0.711331357 | 3.462068219 |
| NAP1L5 | 0.711287309 | 3.155412222 |
| COL26A1 | 0.710986397 | 6.191433562 |
| C15orf65 | 0.710720574 | 2.371486978 |
| LOC100507462 | 0.709482874 | 1.714696621 |
| MRPS34 | 0.709460735 | 7.55233214 |
| MRPL15 | 0.709221098 | 5.347946495 |
| LOC101928764 | 0.708582153 | 2.386774557 |
| TTC32 | 0.708300805 | 4.096280961 |
| UBE3A | 0.707958789 | 6.04960058 |
| 42979 | 0.707553104 | 3.568540296 |
| KCNE2 | 0.70677875 | 1.987809862 |
| GNG7 | 0.706449986 | 3.241523334 |
| HMG20A | 0.705758856 | 5.682692666 |
| HLA-A | 0.70551294 | 3.30332565 |
| EPN1 | 0.705105852 | 6.499748804 |
| POLR2C | 0.704928143 | 5.951414261 |
| PIGL | 0.704789276 | 3.67669749 |
| PPT1 | 0.701706301 | 7.515026501 |
| TNFSF10 | 0.700914071 | 1.44088774 |
| TNFRSF13C | 0.698634315 | 3.193192424 |
| IRAK2 | 0.697447388 | 2.793468433 |
| MOGS | 0.697383126 | 5.992868245 |
| RRBP1 | 0.697064553 | 6.760876215 |
| PLAT | 0.695883469 | 1.123509605 |
| ING4 | 0.69423036 | 4.768300756 |
| VAT1 | 0.694070874 | 7.324075147 |
| HEXIM2 | 0.693589387 | 4.52353337 |
| ALDH3B1 | 0.69319941 | 4.06579985 |
| TCOF1 | 0.692384823 | 5.672612556 |
| PDSS2 | 0.691694369 | 4.554341478 |
| TMEM117 | 0.691553673 | 4.57389257 |
| DHFRL1 | 0.691459298 | 3.391761687 |
| C1orf185 | 0.691153043 | 2.769362709 |
| MANF | 0.68809122 | 7.35644359 |
| DNAJC4 | 0.687931341 | 6.095226755 |
| GPX2 | 0.687759416 | 2.037356454 |
| HBQ1 | 0.687420319 | 4.438278806 |
| OASL | 0.686984633 | 1.70945135 |
| RBP2 | 0.686382837 | 1.969586714 |
| NKX2-8 | 0.684449301 | 1.802783752 |
| TUFT1 | 0.683139963 | 4.326351906 |
| SNCG | 0.68211529 | 6.900498424 |
| RABL6 | 0.681652562 | 7.574829849 |
| ACSM3 | 0.681050608 | 5.060616377 |
| SLC27A3 | 0.679706736 | 3.47938427 |
| BCL2L1 | 0.679429117 | 4.649546715 |
| SAMD10 | 0.678768992 | 1.529014111 |
| UPK2 | 0.678535664 | 2.167177622 |
| FKBP7 | 0.677717774 | 2.343102105 |
| GSTM4 | 0.677521758 | 5.583261057 |
| LYL1 | 0.677398354 | 4.684879362 |
| HERPUD1 | 0.677359278 | 4.784303026 |
| PCNXL3 | 0.677327221 | 4.973980302 |
| CRELD1 | 0.676523129 | 5.052697584 |
| MYH9 | 0.675841429 | 7.458354253 |
| OSTC | 0.675514158 | 6.975337387 |
| CDK9 | 0.67536155 | 5.355694249 |
| PROCA1 | 0.675089297 | 4.757359792 |
| INPP5D | 0.674348996 | 2.451048031 |
| TCF7L1 | 0.67315753 | 4.725433446 |
| LONP2 | 0.67313249 | 5.290901791 |
| C2orf62 | 0.672486283 | 2.929861068 |
| COPS8 | 0.672185998 | 5.711507594 |
| FHIT | 0.671564584 | 3.389734899 |
| STYXL1 | 0.670530124 | 5.020739935 |
| PPP1R9B | 0.670376504 | 6.530398146 |
| WBP11 | 0.668873862 | 6.753562562 |
| HIST1H2AC | 0.667982974 | 4.816678226 |
| CYP2D6 | 0.667924719 | 3.477002463 |
| PIGP | 0.667782189 | 6.134716489 |
| AQP7 | 0.667320991 | 4.196439159 |
| SLC37A4 | 0.666707066 | 5.812888599 |
| SH3PXD2A | 0.665758917 | 4.792633838 |
| ITGB7 | 0.665587949 | 2.430133081 |
| ZNF415 | 0.664402277 | 3.414614372 |
| TPM2 | 0.663580069 | 7.574926898 |
| KLHL12 | 0.663407047 | 6.176225956 |
| LOC102724049 | 0.662145239 | 1.473709743 |
| ZBTB12 | 0.66211736 | 5.303025461 |
| TAMM41 | 0.660459066 | 3.685379967 |
| TRIB3 | 0.660386273 | 4.94854748 |
| SMPD3 | 0.659724735 | 1.305318212 |
| MFN1 | 0.65970159 | 5.695298539 |
| FAM195A | 0.659477605 | 5.919082294 |
| SLC9A3 | 0.659276701 | 3.909686012 |
| EEPD1 | 0.65843973 | 3.052690782 |
| TTC27 | 0.657951584 | 5.190587901 |
| SIAH2 | 0.657278981 | 4.730106306 |
| C19orf45 | 0.656967704 | 3.587520608 |
| BIN3 | 0.656945814 | 4.42804712 |
| SETSIP | 0.656735864 | 2.415248595 |
| REEP5 | 0.656728696 | 5.970860735 |
| SDR39U1 | 0.656376032 | 5.465511828 |
| OSBPL2 | 0.654579358 | 4.364537246 |
| ITGA2B | 0.654297141 | 1.176340618 |
| SH2B1 | 0.653861596 | 5.09550981 |
| ALPK1 | 0.653114042 | 2.487107674 |
| PPP1CB | 0.652291924 | 7.51985999 |
| PPM1H | 0.651918913 | 4.47268266 |
| C2orf15 | 0.651374087 | 3.433264691 |
| HARS | 0.650862672 | 5.319326521 |
| DOCK1 | 0.650467151 | 5.895972428 |
| LOC100129924 | 0.650456683 | 2.348815915 |
| CTTN | 0.650404433 | 7.018345664 |
| FCGBP | 0.650108914 | 2.134624558 |
| C1orf228 | 0.649980385 | 2.707219735 |
| NCAM2 | 0.6489848 | 5.464252951 |
| KIAA1211 | 0.648984795 | 4.248458747 |
| CXCR4 | 0.648941186 | 1.422615757 |
| FAM175A | 0.648906682 | 5.951182272 |
| PMVK | 0.648593932 | 6.060215583 |
| CIR1 | 0.648474151 | 4.810204138 |
| GTF3C6 | 0.648262693 | 6.562902136 |
| IFT52 | 0.647148357 | 5.382160541 |
| RBM15B | 0.64583436 | 5.30902399 |
| TM4SF1 | 0.645600607 | 2.228783251 |
| RGS5 | 0.64555281 | 2.318552334 |
| ZNF622 | 0.644953685 | 4.753600469 |
| FCN3 | 0.644912698 | 1.09855636 |
| CCDC120 | 0.644626247 | 3.485965339 |
| TXLNA | 0.643617054 | 6.208399906 |
| AMACR | 0.643353909 | 3.022052039 |
| STK40 | 0.642657613 | 5.249204056 |
| ISY1 | 0.641681001 | 4.107752318 |
| TRPV2 | 0.641205247 | 2.100346309 |
| AKT1 | 0.640530937 | 6.975652784 |
| GLE1 | 0.639872582 | 5.510486135 |
| PPP1R32 | 0.639326202 | 1.87980541 |
| ZBTB32 | 0.638734484 | 1.906845915 |
| ACY1 | 0.638146985 | 4.408407966 |
| HLA-DMB | 0.638122599 | 3.179170032 |
| EPB41L1 | 0.63787439 | 4.297400052 |
| TEX40 | 0.637825612 | 1.999186352 |
| HIST2H3D | 0.63645864 | 1.431224704 |
| MRPL22 | 0.635924607 | 4.575096658 |
| BBS1 | 0.635791447 | 4.656683575 |
| RAB2A | 0.63549936 | 5.962611947 |
| WBSCR27 | 0.6353342 | 4.676649847 |
| BBC3 | 0.634775314 | 4.689018527 |
| POMGNT1 | 0.634766108 | 5.490513711 |
| LPXN | 0.634756292 | 3.909831915 |
| APEH | 0.633436034 | 6.672277806 |
| NOSIP | 0.631811769 | 5.912691832 |
| ZNF688 | 0.631430275 | 3.541973043 |
| ANXA1 | 0.631404285 | 1.711078575 |
| DDTL | 0.631077746 | 3.446316924 |
| IGFBP4 | 0.63054276 | 3.186740607 |
| RARG | 0.629499069 | 6.50410585 |
| ATF1 | 0.628107332 | 4.8954471 |
| PTPN2 | 0.627628616 | 4.799265179 |
| TRAF3 | 0.626866043 | 4.818761584 |
| CCDC104 | 0.626745993 | 6.921605987 |
| VPS52 | 0.625570458 | 5.339908993 |
| KDM8 | 0.625061791 | 2.517194769 |
| RIBC1 | 0.624185354 | 3.423276561 |
| RTDR1 | 0.623487971 | 2.225044256 |
| TES | 0.623347922 | 1.238894032 |
| LOC100652758 | 0.623334916 | 4.07687491 |
| DIAPH1 | 0.622374112 | 6.417978809 |
| HYOU1 | 0.62229013 | 7.229783197 |
| MTERFD3 | 0.621914447 | 4.790112872 |
| GSTM1 | 0.621864404 | 6.23843522 |
| TAS1R1 | 0.621753718 | 1.33417006 |
| CAMKMT | 0.620963115 | 4.570400141 |
| ADCK4 | 0.620362068 | 5.991834243 |
| AMT | 0.62029543 | 4.289580614 |
| DGKA | 0.620044823 | 5.586422327 |
| CASP10 | 0.619289083 | 1.099399 |
| TOP1 | 0.61910361 | 6.53602958 |
| LDHD | 0.619017555 | 4.107047347 |
| LOC102724747 | 0.618776516 | 3.064307202 |
| ITPA | 0.618082781 | 5.017579589 |
| IER3IP1 | 0.618001954 | 6.51166698 |
| PRKAR1B | 0.617890487 | 3.696105224 |
| LCN12 | 0.617209831 | 3.525549048 |
| OSBPL5 | 0.616576282 | 4.196096564 |
| AAMDC | 0.616116679 | 4.736010552 |
| DDIT4 | 0.616094958 | 8.914057898 |
| COL5A1 | 0.61538356 | 1.795631903 |
| ATF6B | 0.615052603 | 6.642767326 |
| ZSCAN10 | 0.614537868 | 2.130216056 |
| OSBPL11 | 0.614459745 | 4.345604076 |
| PKP2 | 0.61441786 | 2.592574751 |
| HSPA12B | 0.614252048 | 2.154664383 |
| PDCD7 | 0.61362646 | 4.931766965 |
| FMO5 | 0.613584719 | 3.580707155 |
| UBE2D1 | 0.612897504 | 5.146351715 |
| YIPF2 | 0.61252938 | 5.302592098 |
| UPRT | 0.611637869 | 4.472778053 |
| PLA2G3 | 0.611613833 | 6.004424022 |
| RNPC3 | 0.611462184 | 4.88731808 |
| C15orf57 | 0.611217019 | 4.271646829 |
| ZNF182 | 0.610810198 | 3.360773292 |
| HSPD1 | 0.61061463 | 9.592540855 |
| CHRDL1 | 0.610501626 | 3.264113771 |
| PRRC1 | 0.610175657 | 5.687216511 |
| FBXO15 | 0.610155408 | 3.240689082 |
| TMEM141 | 0.609692782 | 6.354787476 |
| KLHDC10 | 0.609374052 | 4.812028671 |
| ACOT1 | 0.609185131 | 2.450618432 |
| RMDN1 | 0.608707774 | 5.466229666 |
| GPR19 | 0.608358714 | 3.242598402 |
| F3 | 0.608346157 | 1.731127622 |
| DNAJC30 | 0.607930382 | 4.126332426 |
| FAHD1 | 0.607100095 | 6.299412771 |
| STAP2 | 0.606899935 | 5.23190239 |
| UBP1 | 0.606748788 | 6.621385089 |
| FAM127B | 0.606712319 | 6.404411283 |
| OCEL1 | 0.606504635 | 5.612689492 |
| NYNRIN | 0.606469798 | 3.434143715 |
| CCNB3 | 0.605251123 | 3.156924834 |
| URGCP | 0.605134896 | 4.344121989 |
| TMEM129 | 0.604760569 | 5.524934303 |
| TEX9 | 0.60465663 | 4.582756603 |
| MESP1 | 0.604650932 | 3.577712253 |
| MTA1 | 0.6045418 | 6.976452659 |
| SFR1 | 0.604334214 | 4.546185327 |
| ZCCHC24 | 0.604109326 | 3.836575934 |
| MEMO1 | 0.60362095 | 6.111134631 |
| RPIA | 0.60349713 | 5.029924835 |
| ARHGEF25 | 0.603295284 | 5.05535582 |
| SAMD15 | 0.603258341 | 2.235336566 |
| NSF | 0.603112803 | 5.352687523 |
| SLC4A1AP | 0.602771158 | 5.872193243 |
| LOC400499 | 0.602692778 | 1.805188544 |
| FAM171A1 | 0.602617265 | 4.616610903 |
| ABHD14B | 0.60245338 | 6.613636598 |
| C1orf233 | 0.60208104 | 5.34223043 |
| NLRP2 | 0.602061577 | 5.031834754 |
| AFG3L2 | 0.601489088 | 6.426602655 |
| HIST1H2AG | 0.601387461 | 3.936703351 |
| HIST1H2BO | 0.601313898 | 1.397117934 |
| SALL2 | 0.600657676 | 5.514179949 |
| MEX3D | 0.600370633 | 5.681418273 |
| TVP23B | 0.60021227 | 5.63153795 |
| NAF1 | 0.600156393 | 4.432806664 |
| C11orf1 | 0.599872374 | 5.860852756 |
| MARCKS | 0.59975874 | 3.037290317 |
| RNF103-CHMP3 | 0.599106358 | 4.553622684 |
| TMEM81 | 0.598365753 | 2.555998555 |
| ULK3 | 0.598316825 | 4.810431446 |
| ZNF251 | 0.59806643 | 5.334727057 |
| PSMB8 | 0.597987626 | 1.163094631 |
| FERMT3 | 0.597928075 | 1.816061643 |
| ACOX2 | 0.597919468 | 1.29816081 |
| P2RX6 | 0.597612148 | 1.831132638 |
| HSD17B14 | 0.597526437 | 6.951149526 |
| PHF7 | 0.597437801 | 3.957950588 |
| CYP1B1 | 0.596895004 | 4.791654617 |
| KARS | 0.596796747 | 7.07673196 |
| TSHZ1 | 0.595891031 | 3.670275029 |
| NFKBIB | 0.595415051 | 4.268754121 |
| ARID5B | 0.595067408 | 1.475615355 |
| C22orf46 | 0.594787216 | 4.161429853 |
| TSPYL4 | 0.594461747 | 5.074919289 |
| TIPRL | 0.594247462 | 6.310025527 |
| RIPK2 | 0.593810326 | 4.06488217 |
| SPCS2 | 0.593746038 | 5.637397891 |
| COPS4 | 0.593745837 | 6.083179637 |
| SYNE4 | 0.593656624 | 6.067366655 |
| MTHFD2L | 0.593616293 | 2.435959303 |
| C2orf57 | 0.593533192 | 1.205965012 |
| NIT1 | 0.592526508 | 5.722102186 |
| EFCAB2 | 0.592474255 | 4.300427338 |
| MYO15A | 0.591765355 | 2.422042839 |
| CGREF1 | 0.591474544 | 5.858148017 |
| TRAPPC6A | 0.590962131 | 4.861665109 |
| ZFP2 | 0.59077975 | 2.187223611 |
| DUSP3 | 0.589958428 | 5.844626126 |
| OR2B6 | 0.589876891 | 1.09551132 |
| ZFP90 | 0.589499255 | 4.604238735 |
| C14orf93 | 0.589329882 | 4.405321337 |
| KIF3C | 0.588529294 | 4.662546854 |
| C11orf84 | 0.588340515 | 5.934611018 |
| GJD3 | 0.587982058 | 2.950113851 |
| XBP1 | 0.587515906 | 6.505610659 |
| UBE2O | 0.587492689 | 5.79351178 |
| WDR63 | 0.587187791 | 3.360509355 |
| NSMCE2 | 0.586627278 | 5.111120284 |
| PLCXD1 | 0.586425991 | 3.817441323 |
| CSK | 0.586277434 | 5.428416842 |
| MXRA8 | 0.585610416 | 4.291348398 |
| CCDC69 | 0.585563668 | 4.350918169 |
| TNFSF12-TNFSF13 | 0.585087937 | 1.880526636 |
| MSRA | 0.585052992 | 2.962292044 |
| SIL1 | 0.584713675 | 5.643278817 |
| TMEM2 | 0.58470835 | 6.903738552 |
| ZC3H8 | 0.584208409 | 4.814739769 |
| DNAJC10 | 0.584120565 | 5.464963373 |
| ATP6V1E2 | 0.583691438 | 4.316602203 |
| TENC1 | 0.583019984 | 4.660493977 |
| C12orf68 | 0.582498976 | 4.054214547 |
| OSBP | 0.582363883 | 5.648676369 |
| INCA1 | 0.581927269 | 3.921234801 |
| ACAT1 | 0.581596372 | 7.44782026 |
| IFT46 | 0.58119506 | 3.871674773 |
| CBR4 | 0.580982501 | 4.930820597 |
| ISOC2 | 0.580348575 | 6.461352272 |
| LRP5L | -0.580714576 | 2.879030896 |
| CNOT6 | -0.580727332 | 5.368405909 |
| IQGAP3 | -0.580992391 | 6.05473482 |
| CTSK | -0.581213039 | 4.592806925 |
| DPYSL5 | -0.58153178 | 4.519746109 |
| SFT2D2 | -0.581539447 | 5.221086822 |
| ATP13A3 | -0.581694932 | 5.754066655 |
| ALOXE3 | -0.582027235 | 1.47201557 |
| RASD2 | -0.582093472 | 1.69864349 |
| C2orf27A | -0.582885002 | 2.537666084 |
| USP31 | -0.583055492 | 2.950023441 |
| MAN1C1 | -0.583343909 | 1.770484148 |
| METRNL | -0.583597156 | 2.342528313 |
| MRPL34 | -0.583738278 | 6.622445423 |
| HIGD1B | -0.584609745 | 1.0413512 |
| TNKS2 | -0.584815862 | 5.65386348 |
| RBM15 | -0.586044568 | 5.574683331 |
| RAD18 | -0.586114176 | 4.129595441 |
| WDFY1 | -0.586621005 | 5.678049446 |
| IGSF9 | -0.586709763 | 1.526525923 |
| SBNO1 | -0.58739282 | 5.524249756 |
| WBSCR22 | -0.588617026 | 6.988840542 |
| TMC7 | -0.588802026 | 2.670916142 |
| ANKRD13C | -0.588804859 | 4.283368952 |
| TBC1D17 | -0.589065185 | 6.270583662 |
| SAMD8 | -0.589240252 | 5.273377202 |
| FOXR1 | -0.589685056 | 1.386412914 |
| MOXD1 | -0.589694632 | 1.793073774 |
| FRYL | -0.591353976 | 5.432455598 |
| CDA | -0.591431961 | 3.114428168 |
| FAM117B | -0.593224201 | 3.658650063 |
| MAP3K7 | -0.593322336 | 5.221004754 |
| C2orf48 | -0.593398008 | 3.257933643 |
| ACVR2B | -0.593716495 | 3.079939494 |
| SIRT1 | -0.593736934 | 5.320232789 |
| PRDX3 | -0.593950376 | 7.935304719 |
| CPT1B | -0.594409907 | 4.099495219 |
| FAM167A | -0.594673279 | 1.59195342 |
| LRIG2 | -0.595101134 | 4.325229165 |
| NUDT10 | -0.595176636 | 3.90125825 |
| ACPL2 | -0.595674068 | 3.099878051 |
| NPIPB4 | -0.595753638 | 2.462334282 |
| ZBTB34 | -0.595758651 | 3.724939402 |
| SAP25 | -0.595846622 | 2.787528713 |
| CHORDC1 | -0.596108935 | 6.924256864 |
| SETD8 | -0.596544894 | 6.03320325 |
| TMEM159 | -0.596967915 | 2.995393411 |
| MAP6 | -0.597322917 | 0.917393353 |
| TMEM150C | -0.598187283 | 3.187283342 |
| CCDC160 | -0.598401607 | 1.105182667 |
| AQP11 | -0.598611661 | 2.881720895 |
| UQCR10 | -0.598823919 | 6.293152735 |
| SORL1 | -0.599073827 | 3.521708407 |
| POLE3 | -0.59954057 | 5.731295293 |
| KLF10 | -0.599637626 | 5.086167208 |
| ABCC8 | -0.599925708 | 2.918692838 |
| GRK6 | -0.600059295 | 5.184198844 |
| CELA2B | -0.600461727 | 1.451914458 |
| SHISA8 | -0.60137877 | 1.947696802 |
| ARHGEF10 | -0.601468061 | 3.835199225 |
| PRSS16 | -0.60156302 | 6.658450647 |
| RC3H1 | -0.602163139 | 3.868048542 |
| PTPN14 | -0.602441179 | 5.299426517 |
| ZNRF3 | -0.603051882 | 3.251701859 |
| TMEM178B | -0.603111428 | 3.35630447 |
| XPO4 | -0.60324539 | 5.048036292 |
| WDR44 | -0.60414864 | 3.894180344 |
| EPB41L4B | -0.605556214 | 2.715166895 |
| HOXB5 | -0.605703786 | 6.704731923 |
| NOVA2 | -0.605817273 | 2.508207374 |
| CHIC1 | -0.606684442 | 3.744923712 |
| C5orf22 | -0.606989922 | 5.038105552 |
| GMFB | -0.607141396 | 5.588161316 |
| HOXB2 | -0.607186058 | 7.339947001 |
| LRP8 | -0.607508233 | 4.05524561 |
| ZNF443 | -0.608258349 | 2.313921307 |
| CDC42EP1 | -0.608535943 | 5.473848918 |
| FAM89A | -0.60858227 | 4.320305545 |
| TCF7 | -0.608619793 | 1.791215559 |
| CPEB3 | -0.608643777 | 3.141929072 |
| NBPF26 | -0.608756744 | 2.718928397 |
| GPR137C | -0.609411715 | 3.621393371 |
| SGTB | -0.610002597 | 2.770993579 |
| OR2AG2 | -0.610046955 | 1.153689477 |
| CCNJ | -0.610047134 | 4.294511893 |
| CASKIN2 | -0.611236583 | 4.639730955 |
| SLC45A1 | -0.611604675 | 1.256119176 |
| TMEM104 | -0.611824113 | 3.477558255 |
| ZNF329 | -0.612617235 | 3.433435345 |
| ARID5A | -0.612623187 | 2.862765455 |
| PIP4K2A | -0.612653434 | 4.341860439 |
| SERTM1 | -0.613761876 | 1.284612472 |
| DPF1 | -0.614378672 | 2.491500393 |
| ANKRD13D | -0.615064772 | 5.648064572 |
| SLC25A17 | -0.61526647 | 4.987612079 |
| AURKAIP1 | -0.615304766 | 7.42837696 |
| BRI3BP | -0.615396211 | 5.951613531 |
| MIER3 | -0.615858805 | 5.038535857 |
| CCDC84 | -0.61607763 | 5.083460365 |
| CCDC47 | -0.616968159 | 6.684479176 |
| ZNF121 | -0.618623031 | 5.285280971 |
| ARL4A | -0.618663109 | 3.284236519 |
| PPP1R14C | -0.618682471 | 2.133457691 |
| PRKD2 | -0.618961472 | 4.967551891 |
| PPP2R1B | -0.619306145 | 5.35647418 |
| SERTAD1 | -0.620408888 | 5.226735378 |
| KCNJ14 | -0.62074951 | 1.983979263 |
| POLN | -0.620766901 | 1.18109394 |
| FAM104A | -0.621074945 | 6.102603235 |
| LOC101928049 | -0.621383186 | 1.734530677 |
| VLDLR | -0.62218103 | 4.709243124 |
| ASNA1 | -0.622288375 | 7.306761548 |
| WEE1 | -0.622525002 | 5.458732635 |
| SYT7 | -0.623046461 | 1.013674445 |
| SESN3 | -0.623484117 | 5.114214556 |
| LOC102724426 | -0.623607732 | 2.076221145 |
| LOC101928485 | -0.623702037 | 1.80884538 |
| RPRD1A | -0.623868387 | 6.162487535 |
| CFP | -0.625359046 | 1.142226908 |
| MYZAP | -0.625388258 | 1.566850561 |
| DCBLD1 | -0.625876329 | 4.418809277 |
| NPIPA2 | -0.626205052 | 1.163652205 |
| SLC7A5 | -0.626880168 | 6.188488314 |
| PDGFB | -0.627210763 | 2.485051694 |
| IRS1 | -0.627325497 | 4.261853947 |
| PHACTR4 | -0.627465195 | 5.355507035 |
| STARD6 | -0.628420016 | 2.55770384 |
| GAL3ST2 | -0.628514554 | 2.207123472 |
| NOXRED1 | -0.62875055 | 1.706369879 |
| FOXC1 | -0.629336036 | 1.961765448 |
| PHAX | -0.63038461 | 5.048876447 |
| PKIA | -0.630520748 | 1.644461338 |
| TRIML1 | -0.630801726 | 0.953583103 |
| DRC1 | -0.631107962 | 0.944364941 |
| PPP6R3 | -0.631183848 | 6.82987544 |
| NFKBIE | -0.631538208 | 3.427551803 |
| ZNF649 | -0.631539302 | 2.331949053 |
| ISL1 | -0.631748088 | 4.776598883 |
| C16orf59 | -0.631844013 | 5.241213758 |
| OTUD4 | -0.63208491 | 5.123981192 |
| POSTN | -0.632156895 | 6.408538455 |
| FAM86KP | -0.633917545 | 1.221397813 |
| ANKRA2 | -0.634325812 | 4.689481344 |
| TMEM86B | -0.634871202 | 3.29230653 |
| TET1 | -0.636393661 | 4.870877134 |
| NACC2 | -0.636553493 | 4.598300306 |
| KIF23 | -0.637388017 | 6.370282717 |
| SHROOM3 | -0.641235894 | 5.592755832 |
| NUFIP2 | -0.641775304 | 6.069820453 |
| LRRC58 | -0.642203847 | 5.726430309 |
| BEND4 | -0.642595314 | 3.541909857 |
| ARVCF | -0.642976987 | 3.573375611 |
| ANKRD34B | -0.643259428 | 3.113420928 |
| NPIPB3 | -0.643422689 | 2.195678762 |
| MICB | -0.644419546 | 5.997672376 |
| HSD11B2 | -0.644468733 | 2.383292351 |
| ONECUT1 | -0.644754711 | 3.675468886 |
| CYP27A1 | -0.644930123 | 1.354429381 |
| PBX4 | -0.644978739 | 2.897680324 |
| PAWR | -0.646300596 | 4.283125252 |
| LYG1 | -0.646345348 | 3.825308515 |
| ZBTB46 | -0.647542659 | 2.745697904 |
| EBPL | -0.64838749 | 6.667606725 |
| FRMD8 | -0.649197009 | 4.301283107 |
| GFOD1 | -0.650647716 | 2.69325745 |
| BRWD1 | -0.652001595 | 4.72975387 |
| VMA21 | -0.652220845 | 4.362498977 |
| ITGA2 | -0.652531181 | 2.640892169 |
| ZNF136 | -0.653446063 | 2.664881251 |
| SMAD6 | -0.65440892 | 4.68747136 |
| USP14 | -0.654611658 | 6.170134628 |
| REV3L | -0.655131131 | 4.999899937 |
| TNFAIP8L1 | -0.655189708 | 4.699164842 |
| NUAK2 | -0.655256581 | 1.080885295 |
| APCDD1 | -0.656091506 | 1.650265726 |
| QRFP | -0.656575691 | 1.647490081 |
| FZD5 | -0.656669352 | 2.720729143 |
| PRELID2 | -0.657255103 | 4.104728287 |
| HIST1H3J | -0.657578006 | 1.500894122 |
| PTGER1 | -0.658316627 | 1.264490911 |
| LAPTM4B | -0.65951323 | 7.76197826 |
| ATF3 | -0.66090341 | 1.08380308 |
| DAGLA | -0.660951531 | 2.248837865 |
| UXS1 | -0.66226649 | 5.617297132 |
| RIMKLB | -0.662512437 | 6.196087384 |
| SCARF1 | -0.663079473 | 1.736247096 |
| PPIF | -0.663487149 | 5.697077881 |
| USP12 | -0.663839761 | 4.9506001 |
| FAM156B | -0.664033991 | 1.045951401 |
| AGO2 | -0.665456818 | 5.287675579 |
| RAD21L1 | -0.665962716 | 1.920267187 |
| ABCA1 | -0.666774495 | 5.050573257 |
| PLEKHF1 | -0.667313374 | 2.689640143 |
| NRBP1 | -0.667895357 | 7.521830375 |
| NXPH4 | -0.667985096 | 2.698029593 |
| LOC100652901 | -0.668072985 | 1.818520648 |
| FAM160B1 | -0.668414533 | 4.62846118 |
| CXXC5 | -0.668842604 | 1.622435939 |
| RMND5A | -0.668884331 | 5.455457423 |
| RHBDL3 | -0.668975452 | 2.328317396 |
| NCEH1 | -0.669104079 | 3.05613926 |
| GABRA5 | -0.67004833 | 3.042237554 |
| GALNT12 | -0.67124649 | 4.624871921 |
| HPSE | -0.671388426 | 2.703208084 |
| CCNE1 | -0.671632119 | 4.587693283 |
| HPCAL4 | -0.671876606 | 3.134917744 |
| GPR180 | -0.672148228 | 4.453788137 |
| VRK3 | -0.672274903 | 5.28878416 |
| IL17D | -0.673045204 | 2.290452869 |
| SPHK1 | -0.67318137 | 3.842761062 |
| SLC19A1 | -0.673883645 | 4.050608889 |
| NPTX2 | -0.673922547 | 3.191556208 |
| DMBX1 | -0.675363228 | 3.774319627 |
| MEGF6 | -0.675829402 | 1.48492037 |
| LAG3 | -0.676174736 | 2.212600156 |
| RHBDD2 | -0.676732849 | 5.645652216 |
| SLC45A4 | -0.676926659 | 2.002650719 |
| ALDH1A3 | -0.677265391 | 3.562971368 |
| TMSB15A | -0.6773612 | 4.624587737 |
| PGM2L1 | -0.677766001 | 1.80730618 |
| ZBTB39 | -0.677885765 | 3.86653688 |
| EGR1 | -0.678256793 | 4.444237995 |
| PSEN2 | -0.678733564 | 4.646717512 |
| TM2D2 | -0.678921701 | 4.596874084 |
| DMWD | -0.679041414 | 4.844032058 |
| RCOR1 | -0.679120636 | 6.47042117 |
| B3GNT5 | -0.679515618 | 3.019655685 |
| TEAD1 | -0.679771753 | 5.358011716 |
| KLF3 | -0.680865387 | 4.354388872 |
| ATP1A3 | -0.680917708 | 1.847981226 |
| SH2B3 | -0.681006981 | 3.493249732 |
| PLAU | -0.681225195 | 3.414681773 |
| UBE2W | -0.681422817 | 3.973347719 |
| ADRB1 | -0.6822539 | 1.765153909 |
| GPC4 | -0.683144437 | 4.035809694 |
| FTL | -0.683267657 | 9.895211373 |
| KPNA2 | -0.683343376 | 9.008982921 |
| MANSC4 | -0.684829834 | 1.132788953 |
| RPS6KA6 | -0.684928626 | 3.591962319 |
| ANKRD46 | -0.685283519 | 4.457014445 |
| QRICH2 | -0.685506565 | 2.799273201 |
| MFAP3L | -0.686826801 | 3.663773468 |
| ELOVL7 | -0.687391399 | 3.440256277 |
| CMTM1 | -0.68769429 | 2.859429591 |
| TST | -0.687767536 | 4.897679482 |
| CMPK1 | -0.689182677 | 6.020624852 |
| ANLN | -0.689476302 | 6.652651067 |
| WNT8B | -0.690889118 | 1.060898097 |
| SLC30A3 | -0.694642022 | 1.921490196 |
| TRAPPC3L | -0.695486856 | 7.203339903 |
| NBPF14 | -0.695766037 | 3.090139035 |
| SHISA9 | -0.696344429 | 1.128979167 |
| HIST2H2BF | -0.69642791 | 1.645257748 |
| KIAA1549 | -0.697029546 | 3.354105553 |
| DAAM1 | -0.697420255 | 3.077917672 |
| POLQ | -0.697572501 | 4.376181666 |
| YY2 | -0.698381784 | 1.662629703 |
| SMAD7 | -0.699898313 | 2.728808581 |
| NUSAP1 | -0.700412365 | 6.851487122 |
| XCR1 | -0.700978749 | 0.958001236 |
| AJUBA | -0.70128141 | 4.486783729 |
| P2RY1 | -0.70238339 | 2.75309041 |
| EFHD1 | -0.703131023 | 5.04873542 |
| SKIL | -0.704436749 | 4.124251752 |
| CROT | -0.704443324 | 3.636786878 |
| AGFG2 | -0.705248032 | 4.595385336 |
| MCF2L2 | -0.705296838 | 1.106351598 |
| GNRH1 | -0.705350618 | 2.731764537 |
| C1QL3 | -0.705680444 | 2.273994004 |
| E2F5 | -0.705937865 | 5.468833588 |
| TERT | -0.707655384 | 4.791235923 |
| LOC101929046 | -0.708680237 | 1.600357966 |
| BMF | -0.709447433 | 2.512536369 |
| GCH1 | -0.710613719 | 3.275010228 |
| SOWAHC | -0.710735492 | 4.216411228 |
| FOXN2 | -0.710965873 | 5.446827058 |
| HAS3 | -0.712207944 | 1.681069005 |
| STX6 | -0.712660964 | 5.685525431 |
| PTPMT1 | -0.713010242 | 4.255680833 |
| C16orf46 | -0.71519683 | 2.056019387 |
| NTN5 | -0.715313745 | 2.576906746 |
| HACE1 | -0.71627722 | 5.569523637 |
| GPR137B | -0.71705292 | 5.321405456 |
| LCLAT1 | -0.717299737 | 4.174808017 |
| LITAF | -0.718030863 | 6.116328987 |
| TMPPE | -0.718362248 | 2.715438524 |
| HAUS2 | -0.718476784 | 4.09309784 |
| PHLPP2 | -0.719315575 | 3.908259743 |
| CYSLTR2 | -0.720061928 | 4.518541342 |
| NBPF10 | -0.720520994 | 2.840119799 |
| SMPD1 | -0.720819355 | 3.509327868 |
| C16orf74 | -0.721165121 | 3.145943895 |
| HOXC8 | -0.721385716 | 4.942495801 |
| SLC12A7 | -0.721680518 | 4.427575865 |
| C2orf69 | -0.722553435 | 4.76286925 |
| ABHD17C | -0.723155689 | 4.152853992 |
| CCDC62 | -0.723241666 | 1.461871849 |
| S100A11 | -0.724341696 | 7.914760307 |
| SFRP1 | -0.726615939 | 4.081230538 |
| PLEKHH2 | -0.727542616 | 2.303342523 |
| VWA5B2 | -0.72758223 | 1.953383267 |
| SPOPL | -0.727948862 | 3.975319176 |
| TGFBR3 | -0.728141212 | 3.076816861 |
| ATP6V0A2 | -0.728832727 | 3.514835099 |
| GALNT7 | -0.729586854 | 4.675178416 |
| SHISA7 | -0.730069342 | 2.160472025 |
| MAP3K3 | -0.730631997 | 4.47727773 |
| HEG1 | -0.730901433 | 1.761349521 |
| FCER1G | -0.731224711 | 1.045746951 |
| PRRT4 | -0.731554481 | 2.233179773 |
| LOC101929561 | -0.733303602 | 1.675537499 |
| TYRO3 | -0.734912296 | 3.840691136 |
| ORAI1 | -0.735160213 | 5.657535045 |
| TGIF1 | -0.737119944 | 6.168692606 |
| NUP50 | -0.737576925 | 5.710089495 |
| GADD45B | -0.739058258 | 4.810833546 |
| PQLC3 | -0.739323362 | 4.963551379 |
| SCG5 | -0.740290129 | 2.039290521 |
| EIF5A2 | -0.740417635 | 3.898588071 |
| 42990 | -0.740423178 | 1.114223939 |
| LOC101929728 | -0.740452504 | 1.856896317 |
| CCR1 | -0.742438806 | 6.362254314 |
| SLC25A30 | -0.743201828 | 4.932936109 |
| WDR62 | -0.743449021 | 5.077624273 |
| ENTPD2 | -0.743625374 | 2.940690927 |
| WHAMM | -0.744011204 | 3.946842165 |
| ART5 | -0.744102055 | 1.739898856 |
| NT5M | -0.744461068 | 3.533947271 |
| C11orf82 | -0.746538726 | 3.917842446 |
| SPSB4 | -0.747244673 | 2.465564588 |
| ID1 | -0.747271227 | 6.81194686 |
| GLTSCR1 | -0.747762375 | 3.947142922 |
| B4GALNT1 | -0.748101569 | 4.373981801 |
| GAD1 | -0.748111576 | 2.678341835 |
| KBTBD2 | -0.748863266 | 4.701388259 |
| OAZ3 | -0.749517372 | 4.353851108 |
| C19orf25 | -0.750898896 | 3.659041483 |
| NBPF19 | -0.751692387 | 3.748786573 |
| NTNG2 | -0.753576097 | 1.288973025 |
| ZNF850 | -0.755721252 | 1.757772425 |
| PNPLA2 | -0.756388748 | 5.981088834 |
| FAM26E | -0.756594148 | 2.675620708 |
| AC004019.13 | -0.756735848 | 1.290136893 |
| SOX8 | -0.757721993 | 2.129890307 |
| MAP3K14 | -0.758180581 | 3.804511002 |
| GID4 | -0.760542696 | 3.979844687 |
| ZNF880 | -0.761886446 | 1.526032206 |
| C9orf117 | -0.763932655 | 2.33857971 |
| HOXB3 | -0.764681602 | 6.865802508 |
| GTF2A1 | -0.765070489 | 5.443646866 |
| PPP2CB | -0.766146772 | 5.856987684 |
| CDK17 | -0.767268809 | 3.981078639 |
| CISH | -0.768193656 | 1.382983862 |
| TMEM245 | -0.769999594 | 5.359759819 |
| CDK5R1 | -0.770094199 | 2.153279696 |
| TMEM79 | -0.771669032 | 3.129988275 |
| RTN4R | -0.77286921 | 3.168174064 |
| ITPR1 | -0.773443779 | 2.700146992 |
| P2RX2 | -0.775967145 | 1.162285769 |
| RABGAP1 | -0.777895711 | 5.62025878 |
| FAM64A | -0.779011476 | 5.195316009 |
| POLR3G | -0.779585928 | 4.396410962 |
| HNRNPK | -0.779840663 | 8.926479188 |
| ZFP69 | -0.783016498 | 1.762621968 |
| CCDC167 | -0.783683376 | 5.227047417 |
| BAIAP3 | -0.784561528 | 3.068122511 |
| SPATA2 | -0.785328541 | 3.57200292 |
| LOC102723893 | -0.785740629 | 1.742351045 |
| CDKN1C | -0.787918585 | 3.346184487 |
| PURG | -0.787985984 | 2.483734009 |
| EVI5 | -0.788040912 | 3.813664558 |
| AFAP1 | -0.789634146 | 1.93242996 |
| MMP17 | -0.790299481 | 2.640365665 |
| MGAT5B | -0.791579225 | 2.170485319 |
| LOC101928991 | -0.793770036 | 2.737250234 |
| IL4R | -0.794545084 | 2.749893879 |
| TNRC6A | -0.794954833 | 5.666280378 |
| ZBTB5 | -0.796930569 | 5.785383359 |
| TMEM191B | -0.798960954 | 1.394308962 |
| SPRY2 | -0.799210065 | 7.238162116 |
| MYH3 | -0.799591138 | 2.619728493 |
| NOXO1 | -0.800246108 | 2.50378459 |
| KCNMB3 | -0.80055269 | 2.148383578 |
| CSPG5 | -0.802223802 | 3.525749933 |
| TPPP | -0.804903223 | 1.957735863 |
| NPAS1 | -0.806408316 | 4.882723668 |
| LOC102724705 | -0.807226525 | 1.190606215 |
| CITED4 | -0.807678955 | 2.384433378 |
| TPPP3 | -0.808610842 | 2.511608569 |
| LOC100505767 | -0.809661189 | 1.730664201 |
| ARPP19 | -0.8102651 | 6.672544418 |
| ZNF331 | -0.811471541 | 4.042963138 |
| NBPF20 | -0.812863707 | 2.797753293 |
| EMR2 | -0.813163949 | 1.659124878 |
| AP1S3 | -0.813698435 | 3.084865989 |
| AMER1 | -0.817812618 | 3.320492203 |
| NT5C2 | -0.821792698 | 6.210818445 |
| FBXO6 | -0.822632823 | 1.394086988 |
| LOC648044 | -0.822763088 | 1.685359637 |
| CELSR2 | -0.827839004 | 4.517516286 |
| GABBR2 | -0.829379194 | 1.637647482 |
| MMP1 | -0.829549002 | 1.431556199 |
| C11orf16 | -0.832090881 | 1.138246471 |
| ANKRD61 | -0.832552928 | 2.795665238 |
| SCGB2B2 | -0.833507016 | 1.721419694 |
| C9orf116 | -0.834803736 | 4.070235139 |
| GIP | -0.835665833 | 2.177005262 |
| IL12A | -0.836356124 | 1.220324422 |
| SNX10 | -0.837276118 | 3.578822628 |
| RASGEF1C | -0.839105492 | 1.585369877 |
| TTC9B | -0.840041617 | 1.241011216 |
| RAB31 | -0.840206928 | 4.017254692 |
| VAMP3 | -0.84277808 | 6.654063154 |
| ANKRD33B | -0.845519028 | 1.19372533 |
| SATB1 | -0.847208318 | 6.827261888 |
| ATP6AP1 | -0.851522142 | 7.051388127 |
| ARL4C | -0.852475732 | 1.386408769 |
| MAP2K4 | -0.854282006 | 5.201913979 |
| NKD2 | -0.85709575 | 2.997554596 |
| SNAI1 | -0.859796828 | 1.70174051 |
| ZYX | -0.862045093 | 4.517863568 |
| SYN2 | -0.863162163 | 3.294005044 |
| LOC102723610 | -0.865115742 | 3.229077758 |
| FAM89B | -0.865383449 | 5.298764868 |
| DUSP2 | -0.868766114 | 2.007008532 |
| CCDC134 | -0.869470281 | 4.322192597 |
| CHRNA4 | -0.869699606 | 2.407087895 |
| CACNA1G | -0.87474807 | 2.76292493 |
| LPAR2 | -0.875009395 | 2.773672075 |
| SOCS5 | -0.875777769 | 5.284345018 |
| SLC4A7 | -0.876347717 | 5.056494168 |
| KLRG2 | -0.877657216 | 2.546454843 |
| HIPK3 | -0.882319366 | 5.254420994 |
| RAET1G | -0.883076798 | 1.512820354 |
| PPP2R3B | -0.883423184 | 3.165854187 |
| IGSF9B | -0.885833593 | 2.92461957 |
| SLC25A18 | -0.886905725 | 1.857411203 |
| FGFR2 | -0.890107629 | 1.565744509 |
| PIM1 | -0.891332504 | 4.842655309 |
| LRRC26 | -0.893564587 | 2.845014697 |
| CYP27B1 | -0.894453482 | 2.533729963 |
| CCND1 | -0.895026433 | 6.516659452 |
| LIN28B | -0.898326326 | 6.115963779 |
| REEP3 | -0.900151494 | 6.888636587 |
| CCNE2 | -0.900796454 | 3.143208093 |
| DLX3 | -0.90108381 | 1.543276592 |
| ARHGAP33 | -0.901597752 | 3.398288568 |
| ADAP1 | -0.902880618 | 2.158491924 |
| SBSPON | -0.90300649 | 1.506435091 |
| LRRC10B | -0.903883032 | 1.360224606 |
| HSPA12A | -0.907409343 | 1.713154956 |
| IGF2BP3 | -0.909223364 | 6.050569819 |
| ZNF221 | -0.909298593 | 2.350830543 |
| DLGAP3 | -0.911270115 | 1.212175402 |
| ZDHHC20 | -0.912219353 | 6.091898992 |
| KPNA3 | -0.912244507 | 5.352580871 |
| PLK5 | -0.912770859 | 1.847156133 |
| KLHL15 | -0.917069212 | 3.6950397 |
| GPR84 | -0.92178867 | 1.487662194 |
| CCND3 | -0.922278251 | 5.811850274 |
| LOC102723572 | -0.927682036 | 2.222689625 |
| RPA4 | -0.92821916 | 1.093704283 |
| CPEB4 | -0.929132425 | 3.674280666 |
| HES1 | -0.931982773 | 4.443629906 |
| COMT | -0.934552144 | 4.920443337 |
| CD70 | -0.936253518 | 2.4774663 |
| PON2 | -0.936498528 | 5.863527533 |
| GNG3 | -0.940543809 | 2.614285031 |
| HOXB8 | -0.940689494 | 6.291011184 |
| RGS10 | -0.944016155 | 6.685776028 |
| LIPA | -0.94415796 | 7.570835169 |
| ZMAT3 | -0.944933935 | 4.167615959 |
| LOC102724336 | -0.947402552 | 3.105362033 |
| SPRY4 | -0.951332754 | 4.928753392 |
| UPP1 | -0.95278609 | 3.998674176 |
| OSTM1 | -0.955559257 | 4.700669774 |
| FEZ1 | -0.956806381 | 1.733587826 |
| SKIDA1 | -0.957903851 | 4.40804375 |
| MCL1 | -0.959232569 | 7.138531422 |
| SH3GL3 | -0.964402446 | 1.294261659 |
| TMEM9B | -0.964798125 | 4.524579686 |
| MDM2 | -0.964910431 | 6.494374696 |
| C1QL1 | -0.972321294 | 4.823918167 |
| FGF18 | -0.973788194 | 6.533795079 |
| PMP22 | -0.975253011 | 2.22479125 |
| EMP1 | -0.977389441 | 9.259598739 |
| FAM212A | -0.979711468 | 2.023077051 |
| CHAC1 | -0.986359471 | 3.645753506 |
| HCN2 | -0.986865348 | 3.281236464 |
| NAA15 | -0.990117893 | 5.426756333 |
| GAN | -0.993909552 | 2.91565058 |
| SH2D7 | -0.996200448 | 1.296303969 |
| ID2 | -0.996930919 | 5.22962979 |
| COX8C | -1.000988594 | 1.179044596 |
| MFSD2A | -1.005247171 | 3.930060235 |
| ITGA6 | -1.006006541 | 5.105086721 |
| ASGR1 | -1.007301151 | 3.353340919 |
| NIPA1 | -1.0151603 | 5.985065448 |
| FAM96B | -1.021329121 | 5.215765432 |
| SRPX | -1.02242051 | 1.823681472 |
| NNAT | -1.024524031 | 1.962180623 |
| YOD1 | -1.028976253 | 4.947240518 |
| GDPD5 | -1.03416881 | 1.628434759 |
| PARD6B | -1.034376677 | 3.799705494 |
| GPR75 | -1.035925896 | 2.446861545 |
| PROZ | -1.042214193 | 1.569626472 |
| CENPQ | -1.050289629 | 3.325458863 |
| LSM11 | -1.05734884 | 3.216799285 |
| IGFBP3 | -1.059165031 | 3.245877647 |
| CCND2 | -1.0607296 | 8.955247993 |
| TMED5 | -1.068771339 | 5.115859037 |
| PSMB9 | -1.0801658 | 2.316012743 |
| SLC6A6 | -1.086894193 | 5.624334491 |
| PITX3 | -1.088646004 | 2.038151325 |
| MORC4 | -1.102282686 | 3.710461522 |
| SCN1B | -1.107163641 | 2.859697249 |
| KIF5C | -1.119628131 | 1.525171748 |
| AMH | -1.124495109 | 5.282719264 |
| DICER1 | -1.133506382 | 5.223954101 |
| RNF38 | -1.133547084 | 4.370762032 |
| GNG13 | -1.137992901 | 4.138212051 |
| GJB5 | -1.144625916 | 1.309154851 |
| E2F7 | -1.14608959 | 3.8643014 |
| ASIP | -1.160218458 | 1.602747917 |
| IGDCC3 | -1.160313602 | 1.628709116 |
| GRIN2C | -1.176055737 | 3.299393466 |
| RASSF2 | -1.179481617 | 1.52048422 |
| CAMKV | -1.20421837 | 1.687147218 |
| S1PR3 | -1.21410811 | 2.817004551 |
| MAB21L1 | -1.214919122 | 7.493585026 |
| ARL2 | -1.247980602 | 6.643497769 |
| SNN | -1.277863152 | 3.307094238 |
| SLC22A20 | -1.28265496 | 2.250846742 |
| DUSP6 | -1.334550413 | 5.325904555 |
| RAB14 | -1.384911352 | 4.987739662 |
| PLLP | -1.473996792 | 3.886467086 |
| MMP3 | -1.486231511 | 1.798984088 |
| UHMK1 | -1.62198397 | 6.082279249 |
| UTF1 | -1.63712212 | 1.876097682 |
| MMP10 | -1.687101219 | 6.380652189 |
| LOC728392 | -1.708780749 | 3.712181316 |
| RBM24 | -1.714991515 | 1.518250604 |
| AMOTL2 | -1.738005895 | 3.868923549 |
