# Supplementary material for: HNRNPH1-stabilized LINC00662 promotes ovarian cancer progression by activating the GRP78/p38 pathway
Source: Oncogene. 2021 Jun 19;40(29):4770–82. doi: 10.1038/s41388-021-01884-5 (PMC8298204; doi:10.1038/s41388-021-01884-5)
Supplement: Supplementary file 5 — Supplementary Table S4 [file 41388_2021_1884_MOESM5_ESM.docx]

**Supplementary Table S4. Results of GSEA enrichment profiles affected by LINC00662 knockdown**

| Description | setSize | NES | pvalue | qvalues |
| --- | --- | --- | --- | --- |
| KEGG_SYSTEMIC_LUPUS_ERYTHEMATOSUS | 72 | -2.21 | 0.002 | 0.044 |
| KEGG_ARACHIDONIC_ACID_METABOLISM | 26 | -1.96 | 0.002 | 0.044 |
| KEGG_TGF_BETA_SIGNALING_PATHWAY | 68 | 1.78 | 0.002 | 0.044 |
| KEGG_GLIOMA | 55 | 1.74 | 0.002 | 0.044 |
| KEGG_ADHERENS_JUNCTION | 62 | 1.83 | 0.002 | 0.044 |
| KEGG_NEUROACTIVE_LIGAND_RECEPTOR_INTERACTION | 80 | 1.83 | 0.002 | 0.044 |
| KEGG_MAPK_SIGNALING_PATHWAY | 203 | 1.55 | 0.003 | 0.044 |
| KEGG_ECM_RECEPTOR_INTERACTION | 55 | -1.72 | 0.003 | 0.044 |
| KEGG_PROPANOATE_METABOLISM | 29 | -1.79 | 0.004 | 0.044 |
| KEGG_METABOLISM_OF_XENOBIOTICS_BY_CYTOCHROME_P450 | 24 | -1.87 | 0.004 | 0.044 |
| KEGG_DRUG_METABOLISM_CYTOCHROME_P450 | 23 | -1.90 | 0.004 | 0.044 |
| KEGG_ALPHA_LINOLENIC_ACID_METABOLISM | 9 | -1.75 | 0.004 | 0.044 |
| KEGG_LINOLEIC_ACID_METABOLISM | 8 | -1.82 | 0.004 | 0.044 |
| KEGG_TYPE_II_DIABETES_MELLITUS | 33 | 1.83 | 0.005 | 0.044 |
| KEGG_MELANOMA | 50 | 1.66 | 0.005 | 0.044 |
| KEGG_JAK_STAT_SIGNALING_PATHWAY | 84 | 1.69 | 0.005 | 0.044 |
| KEGG_OOCYTE_MEIOSIS | 94 | 1.56 | 0.005 | 0.044 |
| KEGG_NITROGEN_METABOLISM | 17 | -1.92 | 0.005 | 0.045 |
