# Supplementary material for: HNRNPH1-stabilized LINC00662 promotes ovarian cancer progression by activating the GRP78/p38 pathway
Source: Oncogene. 2021 Jun 19;40(29):4770–82. doi: 10.1038/s41388-021-01884-5 (PMC8298204; doi:10.1038/s41388-021-01884-5)
Supplement: Supplementary file 6 — Supplementary Table S5 [file 41388_2021_1884_MOESM6_ESM.docx]

| **Supplementary Table S5. Correlation between LINC00662 and clinicopathological parameters in patients with ovarian cancer** | | | | | | | | |
| --- | --- | --- | --- | --- | --- | --- | --- | --- |
| Variables | | n (%) | Expression of LINC00662 | | | χ^2^ | | P value |
|  |  |  | Low | | High |  |  |  |
| Age (years) | |  |  |  | | 1.084 | | 0.298 |
| ≤ 55 | | 37 (49.3) | 21 | 16 | |  | |  |
| > 55 | | 38 (50.7) | 17 | 21 | | |  |  |
| Size (cm) | |  |  |  | | | 0.004 | 0.948 |
| ≤ 5 | | 57(76.0) | 29 | 28 | | |  |  |
| > 5 | | 18 (24.0) | 9 | 9 | | |  |  |
| FIGO stage | |  |  |  | | | 14.427 | 0.000* |
| Ⅰ-Ⅱ | | 26 (34.7) | 21 | 5 | | |  |  |
| Ⅲ–Ⅳ | | 49 (65.3) | 17 | 32 | | |  |  |
| Poor histologic differentiation | |  |  |  | | | 0.644 | 0.422 |
| Yes | | 35 (46.7) | 16 | 19 | | |  |  |
| No | | 40 (53.3) | 22 | 18 | | |  |  |
| Vascular invasion | | |  |  | | | 6.134 | 0.013* |
| Yes | 28 (37.3) | | 9 | 19 | | |  |  |
| No | 47 (62.7) | | 29 | 18 | | |  |  |
| Lymphatic metastasis | | |  |  | | | 2.315 | 0.128 |
| Yes | 28 (37.3) | | 18 | 10 | | |  |  |
| No | 47 (62.7) | | 20 | 27 | | |  |  |
| Distant metastasis | | |  |  | | | 4.808 | 0.028* |
| Yes | 37 (49.3) | | 14 | 23 | | |  |  |
| No | 38 (50.7) | | 24 | 14 | | |  |  |
