# Supplementary material for: HNRNPH1-stabilized LINC00662 promotes ovarian cancer progression by activating the GRP78/p38 pathway
Source: Oncogene. 2021 Jun 19;40(29):4770–82. doi: 10.1038/s41388-021-01884-5 (PMC8298204; doi:10.1038/s41388-021-01884-5)
Supplement: Supplementary file 7 — Supplementary Table S6 [file 41388_2021_1884_MOESM7_ESM.docx]

**Supplementary Table S6. Primers and siRNA sequence used in this study.**

|  | | |  |
| --- | --- | --- | --- |
| **Primers used for quantitative RT-PCR** | | |  |
| **Name** | **Forward-primer** | | **Reverse-primer** |
| LINC00662 | TTTGCCACACTCCTTCAGAGATGCG | | CGTGGCAGGCCAGAGATAGACAAAA |
| GRP78 | GAACGTCTGATTGGCGATGC | | GAGTCGAGCCACCAACAAGA |
| HNRNPH1 | GCAGAACTCGCACAAGGGA | | TCCGGCGTCGAAACAAACTA |
| β-actin | TCCTCTCCCAAGTCCACACA | | GCACGAAGGCTCATCATTCA |
| U6 | CTCGCTTCGGCAGCACA | | AACGCTTCACGAATTTGCGT |
| PGC | AGTCTATCCGTGAGACCATGAA | | GCGGTACTTCCAAGCAGGA |
| LYZ | TCAATAGCCGCTACTGGTGTA | | ATCACGGACAACCCTCTTTGC |
| MUC6 | CTGCCCTATACCAGCAATGGA | | CTGACCCATGTACTTCCGCTC |
| KRT8 | CAGAAGTCCTACAAGGTGTCCA | | CTCTGGTTGACCGTAACTGCG |
| COL1A2 | GAGCGGTAACAAGGGTGAGC | | TTCCCCATTAGGGCCTCTC |
| AMOTL2 | GCTCGTTGAGTGAACGGCT | | CATGAGCTAGTACAACATGAGGG |
| RBM24 | AACCTGGCATACTTAGGAGCA | | AGGTCTTTGTATAAGGGCTGGA |
| LOC728392 | CTGCTATCTCGACCATTTTCCG | | CGATGGCTGCGTAGATGAAG |
| MMP10 | TGCTCTGCCTATCCTCTGAGT | | TCACATCCTTTTCGAGGTTGTAG |
| UTF1 | CGCCGCTACAAGTTCCTTAAA | | GGATCTGCTCGTCGAAGGG |
| **Primers used for RACE-PCR** | |  |  |
| **Name** | | **Sequence** |  |
| LINC00662-5’RACE-GSP | | CTGGAGATGGAAGGAGGATG |  |
| LINC00662-5’RACE-NGSP | | TGCCTAAATAGGACTTTCTTTGC |  |
| LINC00662-3’RACE-GSP | | TATTTAGCACACATTTAATGACAGAAGTC |  |
| LINC00662-3’RACE-NGSP | | GAAGTCTCAGGCTGGAAACACTCTT |  |
| **Primers used for LINC00662 RNA pulldown** | | |  |
| **Name** | **Sequence** | |  |
| LINC00662-sense-F | TAATACGACTCACTATAGGTTTGCCACACTCCTTCAGA | |  |
| LINC00662-sense-R | CAGTTTTTCAGTGTAGGA | |  |
| LINC00662-anti-sense-F | TTTGCCACACTCCTTCAGA | |  |
| LINC00662-anti-sense-R | TAATACGACTCACTATAGGCAGTTTTTCAGTGTAGGA | | |
| **Primers used for LINC00662 deletion mapping** | | |  |
| **Name** | **Forward-primer** | | **Reverse-primer** |
| LINC00662-Exon1 | TAATACGACTCACTATAGGTTTGCCACACTCCTTCAG | | CTTGGAAATCAATTAAAT |
| LINC00662-Exon2 | TAATACGACTCACTATAGGGTGTCCATCGCGCTTCC | | CTGAGACTTCTGTCATTA |
| LINC00662-Exon1+2 | TAATACGACTCACTATAGGTTTGCCACACTCCTTCAGA | | CTGAGACTTCTGTCATTA |
| LINC00662-Exon3 | TAATACGACTCACTATAGGTCTCAGGCTGGAAACACTCT | | CTGGTGAATCAAAACTCAGG |
| LINC00662-Exon3+4 | TAATACGACTCACTATAGGTCTAAGCACATTGAGGCC | | GTAGGACTTAATGGCCTC |
| LINC00662-Exon2+3+4 | TAATACGACTCACTATAGGGTGTCCATCGCGCTTCC | | CAGTTTTTCAGTGTAGGA |
| **Sequence for siRNA** | **Sense (5’-3’)** | |  |
| LINC00662-siRNA1 | GCUGCUGCCACUGUAAUAATT | |  |
| LINC00662-siRNA2 | GCCACGGCUCUGACUUGUUTT | |  |
| LINC00662-siRNA3 | GCCACUGUAAUAAAGCGUGTT | |  |
| GRP78-siRNA1 | GGUGGGCAAACAAAGACAUTT | |  |
| GRP78-siRNA2 | GGUACUGCUUGAUGUAUGUTT | |  |
| GRP78-siRNA3 | GCCACCAAGAUGCUGACAUTT | |  |
| HNRNPH1-siRNA1 | GCUCAAGGUAUUCGUUUCATT | |  |
| HNRNPH1-siRNA2 | CACGAAAGCUUAUGGCCAUTT | |  |
| HNRNPH1-siRNA3 | GGAUUACCUUACAGAGCUATT | |  |
| Si-Control (NC) | UUCUCCGAACGUGUCACGUTT | |  |
