# Supplementary material for: HNRNPH1-stabilized LINC00662 promotes ovarian cancer progression by activating the GRP78/p38 pathway
Source: Oncogene. 2021 Jun 19;40(29):4770–82. doi: 10.1038/s41388-021-01884-5 (PMC8298204; doi:10.1038/s41388-021-01884-5)
Supplement: Supplementary file 8 — Supplementary Table S7 [file 41388_2021_1884_MOESM8_ESM.docx]

**Supplementary Table S7. Antibodies used in this study.**

| **Name** | **Company** | **Catalog Number** | **Assay** |
| --- | --- | --- | --- |
| p27 | Cell Signaling Technology | 3686 | WB |
| Cyclin E | Cell Signaling Technology | 20808 | WB |
| E-cadherin | Cell Signaling Technology | 3195 | WB |
| N-cadherin | Cell Signaling Technology | 13116 | WB |
| MMP9 | Cell Signaling Technology | 13667 | WB |
| Vimentin | Cell Signaling Technology | 5741 | WB |
| HNRNPH1 | Abcam | ab10374 | RIP,WB |
| GRP78 | Proteintech | 11587-1-AP | RIP,WB, IHC |
| P38 | Santa Cruz Biotech | sc-7972 | WB |
| p38 (Tyr182) | Santa Cruz Biotech | sc-166182 | WB, IHC |
| c-Src | Cell Signaling Technology | #2108 | WB |
| c-Src (Tyr419/Tyr424) | Santa Cruz Biotech | sc-81521 | WB |
| AMFR | Proteintech | 16675-1-AP | IP, WB |
| β-actin | Sigma-aldrich | #A3854 | WB |
| Ubiquitin | Cell Signaling Technology | #3936 | WB |
